# Multiscale genetic architecture of donor-recipient differences reveals intronic *LIMS1* mismatches associated with kidney transplant survival

**DOI:** 10.1172/JCI170420

**Published:** 2023-11-01

**Authors:** Zeguo Sun, Zhongyang Zhang, Khadija Banu, Ian W. Gibson, Robert B. Colvin, Zhengzi Yi, Weijia Zhang, Bony De Kumar, Anand Reghuvaran, John Pell, Thomas D. Manes, Arjang Djamali, Lorenzo Gallon, Philip J. O’Connell, John Cijiang He, Jordan S. Pober, Peter S. Heeger, Madhav C. Menon

**Affiliations:** 1 Division of Nephrology, Department of Medicine,; 2 Department of Genetics and Genomic Science, and; 3 Icahn Institute for Data Science and Genomic Technology, Icahn School of Medicine at Mount Sinai, New York, New York, USA.; 4 Department of Medicine, Yale University School of Medicine, New Haven, Connecticut, USA.; 5 Max Rady college of Medicine, University of Manitoba, Winnipeg, Manitoba, Canada.; 6 Harvard Medical school, Boston, Massachusetts, USA.; 7 Yale Center for Genomics, New Haven, Connecticut, USA.; 8 Maine Medical Center, Portland, Maine, USA.; 9 Feinberg School of Medicine, Northwestern University, Chicago, Illinois, USA.; 10 The Westmead Institute for Medical Research, University of Sydney, New South Wales, Australia.; 11 Cedars-Sinai Medical Center, Los Angeles, California, USA.

**Keywords:** Genetics, Transplantation, Adaptive immunity, Genetic variation, Organ transplantation

## Abstract

Donor-recipient (D-R) mismatches outside of human leukocyte antigens (HLAs) contribute to kidney allograft loss, but the mechanisms remain unclear, specifically for intronic mismatches. We quantified non-HLA mismatches at variant-, gene-, and genome-wide scales from single nucleotide polymorphism (SNP) data of D-Rs from 2 well-phenotyped transplant cohorts: Genomics of Chronic Allograft Rejection (GoCAR; *n* = 385) and Clinical Trials in Organ Transplantation-01/17 (CTOT-01/17; *n* = 146). Unbiased gene-level screening in GoCAR uncovered the *LIMS1* locus as the top-ranked gene where D-R mismatches associated with death-censored graft loss (DCGL). A previously unreported, intronic, *LIMS1* haplotype of 30 SNPs independently associated with DCGL in both cohorts. Haplotype mismatches showed a dosage effect, and minor-allele introduction to major-allele-carrying recipients showed greater hazard of DCGL. The *LIMS1* haplotype and the previously reported *LIMS1* SNP rs893403 are expression quantitative trait loci (eQTL) in immune cells for *GCC2* (not *LIMS1*), which encodes a protein involved in mannose-6-phosphase receptor (M6PR) recycling. Peripheral blood and T cell transcriptome analyses associated the *GCC2* gene and *LIMS1* SNPs with the TGF-β1/SMAD pathway, suggesting a regulatory effect. In vitro *GCC2* modulation impacted M6PR-dependent regulation of active TGF-β1 and downstream signaling in T cells. Together, our data link *LIMS1* locus D-R mismatches to DCGL via *GCC2* eQTLs that modulate TGF-β1–dependent effects on T cells.

## Graphical abstract

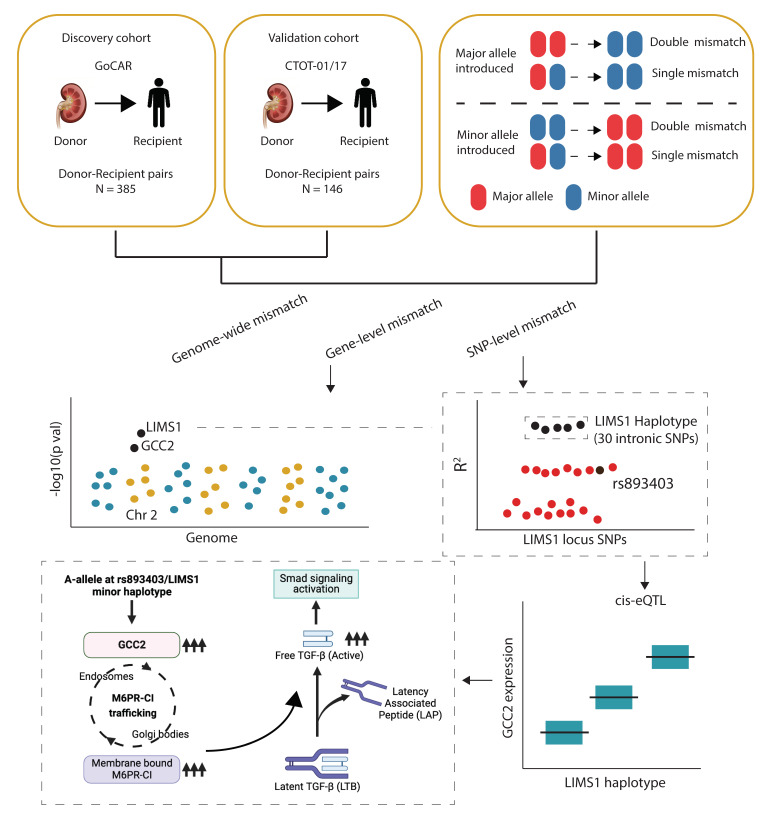

## Introduction

In renal transplantation, short-term allograft outcomes, including acute rejection, have significantly improved, without a proportionate improvement in long-term allograft outcomes (1). While distinct etiologies are identifiable in half of all allograft loss cases, allograft fibrosis or interstitial fibrosis and tubular atrophy (IF/TA) of unclear etiology account for 30% to 40% of cases (2, 3). A role for antidonor alloimmunity is directly implicated in all cases of allograft failure from rejection, but is also reported in IF/TA (without rejection) by transcriptomic data (4). Thus, a majority of allograft loss is related to immune injury.

Alloimmune responses leading to allograft damage are triggered by recipient T cells directly interacting with mismatched, polymorphic donor human leukocyte antigen (HLA) molecules and/or by indirectly recognizing donor-derived peptides processed and presented by recipient HLA on recipient antigen-presenting cells. These cognate interactions are driven by DNA sequence mismatch between HLA regions of donor and recipient on chromosome 6. Non-HLA loci that contribute to allograft rejection can alter protein sequences that, if mismatched between donor and recipient, can trigger activation of pathogenic, donor-reactive T cells in donor-recipient (D-R) pairs that share HLA, and have been traditionally labeled as “minor” histocompatibility (mH) antigens. Our group among others have employed genome-wide screening to advance this field by demonstrating that such D-R genetic differences can impact allograft survival either via rejection (5–7) or via allograft IF/TA (8) independently of HLA matching. Efforts to unravel specific non-HLA gene loci, wherein D-R mismatches disproportionately contribute to transplant outcomes, and understand mechanisms thereof, have clinical applications for allograft risk stratification, surveillance, or even therapeutics. One example is the reported role of mismatches at rs893403 within the *LIMS1* gene locus, a copy number variant–tagging (CNV-tagging), intronic single nucleotide polymorphism (SNP), that was associated with rejection episodes in 3 independent cohorts (9). An increased risk of rejection was observed only when the A allele at this locus was introduced by the donor into G allele–carrying recipients, implying the “directionality” of this allele mismatch.

To provide a more in-depth understanding, herein we undertook an unbiased examination of genome-wide SNP array data from D-R pairs in 2 prospective kidney transplant cohorts — Genomics of Chronic Allograft Rejection (GoCAR) and Clinical Trials in Organ Transplantation-01/17 (CTOT-01/17) (10, 11) — with the goal of identifying mismatches within non-HLA loci that associate with long-term death-censored graft loss (DCGL). After confirming that global D-R genetic differences resulting from SNP mismatches associate with DCGL, we systematically scanned mismatches across all annotated gene loci, to identify individual gene-level mismatches that significantly associated with increased risk of graft loss, independently of HLA. This screening confirmed *LIMS1* as a top-ranked gene locus associated with DCGL, independent of genome-wide mismatches. When we further screened SNP-wise D-R mismatches, we identified 30 SNPs in high linkage disequilibrium (LD), distinct from the previously reported rs893403, which are significantly associated with increased risk of graft loss and demonstrated “directionality” when grouped as haplotype alleles. Our analysis of multiple transcriptomic data sets showed a role for both rs893403 and these linked SNPs as *cis*-expression quantitative trait loci (*cis*-eQTL) for *GCC2*, a gene adjacent to *LIMS1*, that promotes transforming growth factor β1 (TGF-β1, gene *TGFB1*) signaling in lymphocytes/epithelial cells by regulating the trafficking of mannose-6-phosphate receptors (M6PRs), which cleave the latency-associated peptide (LAP) and activate TGF-β1.

## Results

### Description of study cohorts and D-R mismatches

We employed the subsets of kidney transplant D-R pairs with genome-wide genotype information from their parent studies, GoCAR (11) and CTOT-01/17 (10, 12), respectively, as our discovery and validation cohorts in the current study. Demographic and clinicopathologic characteristics of the D-R pairs in the GoCAR (discovery) and CTOT-01/17 (validation) cohorts are shown in Supplemental Table 1 (supplemental material available online with this article; https://doi.org/10.1172/JCI170420DS1) and reported in detail elsewhere (8, 13).

#### Discovery cohort.

Briefly, the parent GoCAR study was a prospective, multicenter study. Enrolled patients had clinical data, longitudinal blood draws, and serial surveillance biopsies collected at 0, 3, 12, and 24 months, detailed in our published studies (8, 11, 14). Genome-wide genotype data were generated for 385 D-R pairs from the GoCAR study followed by an imputation based on the reference panel from the 1000 Genomes Project (Methods). In the GoCAR cohort, the median follow-up time was 1,824 days, 194 (50.4%) were live donors, and 50 (13.0%) DCGL events were observed (Supplemental Table 1 and Methods). The single most common etiology of late DCGL was chronic allograft injury, and over half of all graft losses were related to immunologic events (Supplemental Table 2). There were 36 cases of subclinical or clinical rejection (acute rejection, AR) identified within 2 years of transplant, as described previously (13). After excluding variants with low confidence (INFO score < 0.4) or high missing rate (≥0.05), with no alternative allele, or in the MHC region, there remained 30,109,467 variants to calculate genome-wide D-R mismatch score; after filtering variants with low mismatch frequency (≤0.05), there remained 2,251,582 common variants used for gene-level and variant-level mismatch calculation (Supplemental Figure 1A and Methods).

#### Validation cohort.

Briefly, the parent CTOT-01 study was a prospective, multicenter, observational study that enrolled 280 adult and pediatric, crossmatch-negative kidney transplant candidates. All CTOT-01 recipients were followed up for 2 years after transplantation, while CTOT-17 was designed to observationally collect data on 5-year outcomes among patients in this cohort (Methods). Genome-wide genotype data were generated and imputed as well with the 1000 Genomes Project reference panel for 146 D-R pairs from the parent CTOT-01/17 study (Methods). In the CTOT cohort, the median follow-up time was 1,825 days, 124 (84.8%) were living donors, and 9 (6.2%) DCGL events were observed (Supplemental Table 1 and Methods). The etiologies of graft loss in CTOT have been reported previously (10). The most common cause was chronic allograft injury/chronic rejection. As compared with the GoCAR cohort, the CTOT cohort had younger donors and recipients, fewer rejection and DCGL events, more donors with African American ancestry, and higher HLA mismatch score, while other relevant variables had no significant difference (Supplemental Table 1). With similar quality control steps as GoCAR on the imputed genotype data, a total of 11,091,731 variants remained for genome-wide D-R mismatch score calculation, and 137,777 SNPs were left for the gene-level and variant-level mismatch analyses (Supplemental Figure 1B and Methods).

#### Defining D-R mismatches at different genomic scales.

**Figure 1:**
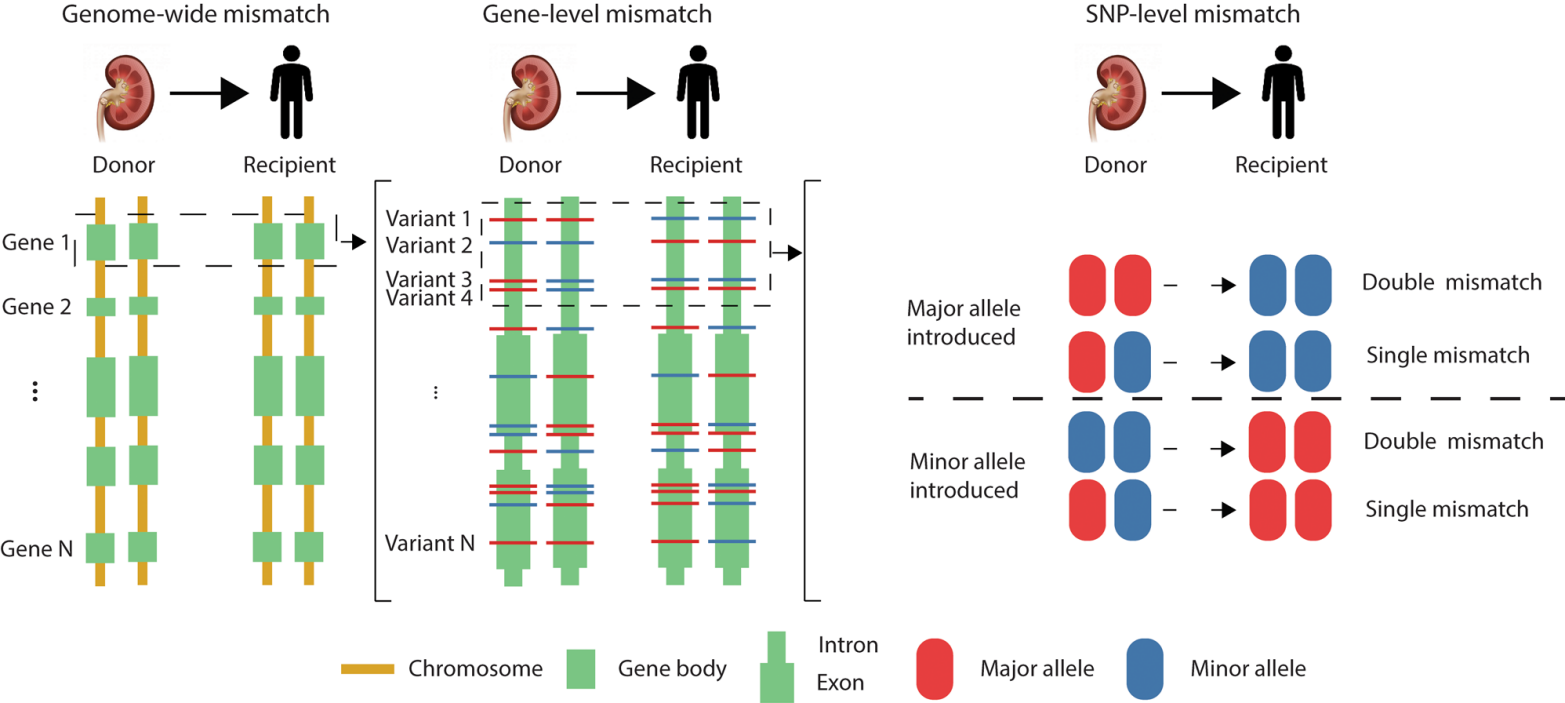
Definition of genetic mismatch between donor-recipient (D-R) pairs at multiple scales. Genetic D-R mismatches are defined at genome-wide (left), gene (middle), and variant (right) levels, followed by association analysis with transplant outcomes. For a specific variant (e.g., SNP), 2 different types of mismatches, single mismatch and double mismatch, are defined as shown in the right side of the diagram. All the double mismatches and single mismatches were defined together as “any mismatch.”

In any given D-R pair in either cohort, we defined 3 levels of donor-to-recipient mismatches based on imputed genotype data: genome-wide mismatches, gene-level mismatches, and variant-level mismatches, where the former 2 were themselves defined based on cumulative variant-level mismatches (Figure 1 and Methods). At the variant level (including SNPs and small indels), a D-R mismatch was defined as the donor carrying 1 or 2 alleles that are not present in the recipient. Depending on the number of “alien” alleles introduced from the donor to the recipient, a variant-level mismatch was further categorized as single mismatch (1 allele introduced), double mismatch (2 alleles introduced), and any mismatch (1 or 2 alleles introduced) (Figure 1). We identified 1,280,475 ± 335,138 of any mismatches in the 385 GoCAR D-R pairs and 233,365 ± 97,270 in the 146 CTOT D-R pairs at the genome-wide scale. The non-exonic region contributed dominantly to the genome-wide mismatch in both cohorts and the nonsynonymous SNPs contributed approximately 50% of the mismatches in exonic regions (Supplemental Table 2). The differences in the total number of mismatches between the 2 cohorts are due to different genotyping platforms and imputation methods, but after normalizing with interquartile range (IQR), the genome-wide mismatch score is consistent between studies (see results below).

### Normalized genome-wide non-HLA D-R mismatch scores are associated with graft loss

**Figure 2:**
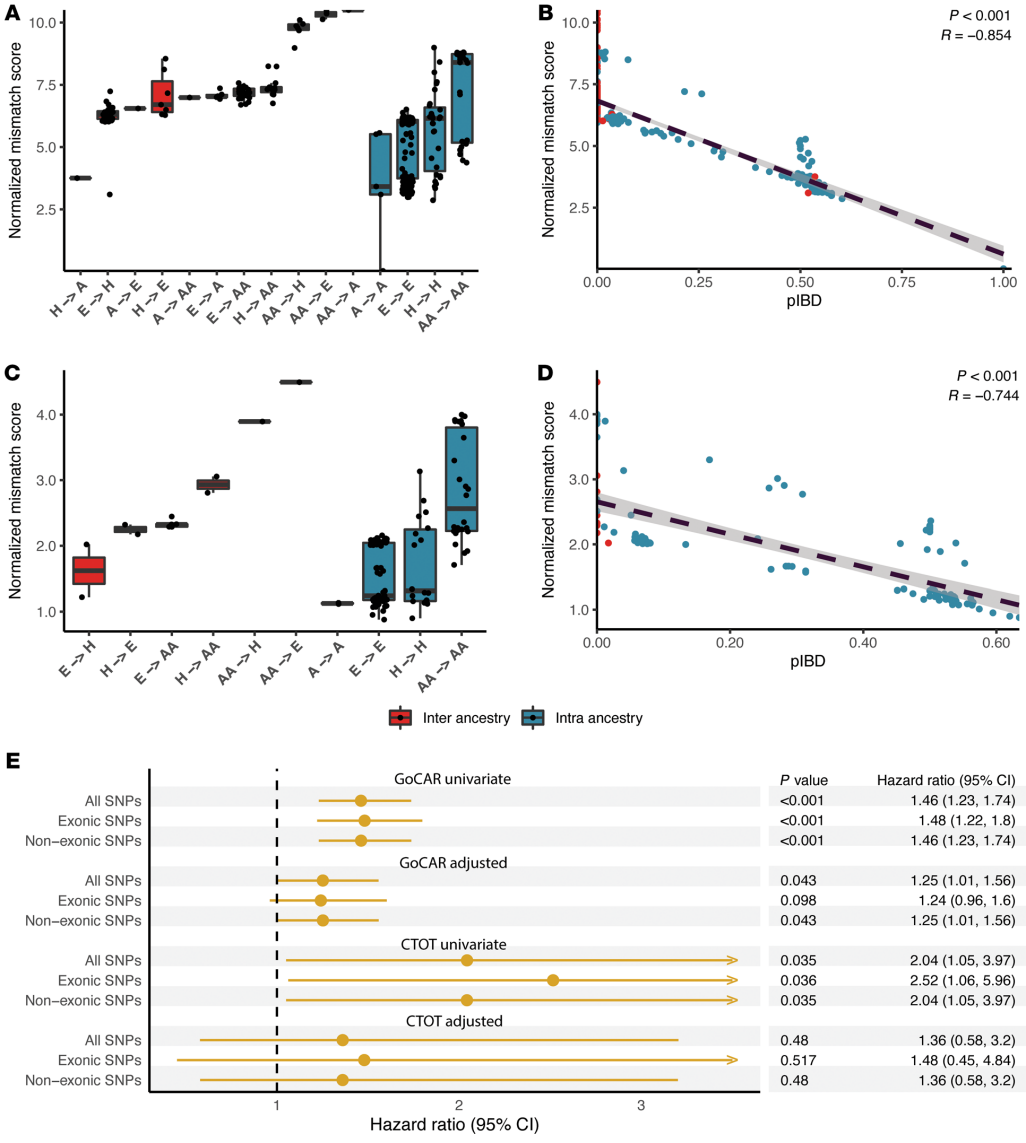
Genome-wide D-R mismatch score was associated with graft loss. Genome-wide mismatch score between donor-recipient (D-R) pairs was calculated at all imputed SNPs with high confidence after quality control, and normalized by IQR. The distribution of normalized genome-wide mismatch scores is shown for D-R pairs stratified by different combinations of D-R genetic ancestries in GoCAR (**A**) and CTOT (**C**), with inter-ancestry D-R pairs in red and intra-ancestry in blue. Genome-wide D-R mismatch score is highly correlated with interpersonal relatedness, reflected as the proportion of identity-by-descent (pIBD) in GoCAR (**B**) and CTOT (**D**). (**E**) Forest plots show the association with DCGL of genome-wide D-R mismatch scores calculated at all imputed SNPs as well as the SNPs within exonic and non-exonic regions. The association analyses were performed using univariate and multivariable Cox regression models adjusted for HLA mismatches, induction therapy, and donor status for both the GoCAR and CTOT cohorts.

We first calculated the normalized genome-wide non-HLA D-R mismatch score (or simply, the genome-wide mismatch score) by summing the variant-level any mismatch scores (0 for match and 1 for mismatch) at all the imputed, quality-controlled SNPs across the genome (excluding the MHC region) as raw mismatch counts. We normalized the raw counts by their IQR for each D-R pair (Methods) (5). This normalized score was able to capture information from both quantitative measures of genome-wide mismatches that we described in previous work: (a) genetic ancestry and (b) proportion of genome-shared identity by descent (pIBD), which are themselves mutually orthogonal (8). First, the normalized mismatch score could reflect the relative differences in genetic ancestries of D-R pairs in both GoCAR and CTOT cohorts, where inter-ancestry pairs generally had larger mismatch scores than intra-ancestry pairs (Figure 2, A and C). Within inter- and intra-ancestry D-R pairs, the distribution of the scores is also consistent with existing knowledge about the relative distance between different major ancestral populations (8). Second, independent of genetic ancestry in both cohorts, the normalized mismatch score was highly correlated with pIBD, a quantitative measure of kinship between 2 individuals (Figure 2, B and D). Hence, we used this variable as a representative quantification of genome-wide D-R mismatches in all association analyses with DCGL. In the discovery cohort, using univariate and multivariable Cox regression (adjusting HLA mismatch score, induction therapy, and donor status), increased genome-wide mismatch scores were associated with DCGL (HR = 1.46, *P* < 0.001 and HR = 1.25, *P* = 0.04, respectively) (Figure 2E). The association in the univariate model was validated in the CTOT cohort, and although we observed a similar HR in the multivariable model, it was less significant, with a wider confidence interval due to lower sample size and event rate (Figure 2E). As sensitivity analyses, we evaluated European-to-European (E-to-E) D-R pairs and identified similar association signals (Supplemental Figure 2). Similarly, we incorporated HLA-DQ mismatches (in GoCAR) and HLA-C, DP, and DQ mismatches (in CTOT) in multivariable Cox models and identified a similar relationship of genome-wide mismatches with DCGL (Supplemental Figure 3).

Within our cohorts, we also aimed to evaluate genome-wide mismatches defined within transmembrane nonsynonymous SNPs versus other genomic regions and tested their reported association with DCGL (Methods) (5). We observed high correlations between these different components of the normalized genome-wide mismatch score (Supplemental Figure 4). Hence, the specific role of transmembrane nonsynonymous SNP mismatches and their impact on graft survival could not be dissected in our data sets.

### Screening of gene-level mismatches unraveled mismatches at the LIMS1 locus associated with graft loss

**Figure 3:**
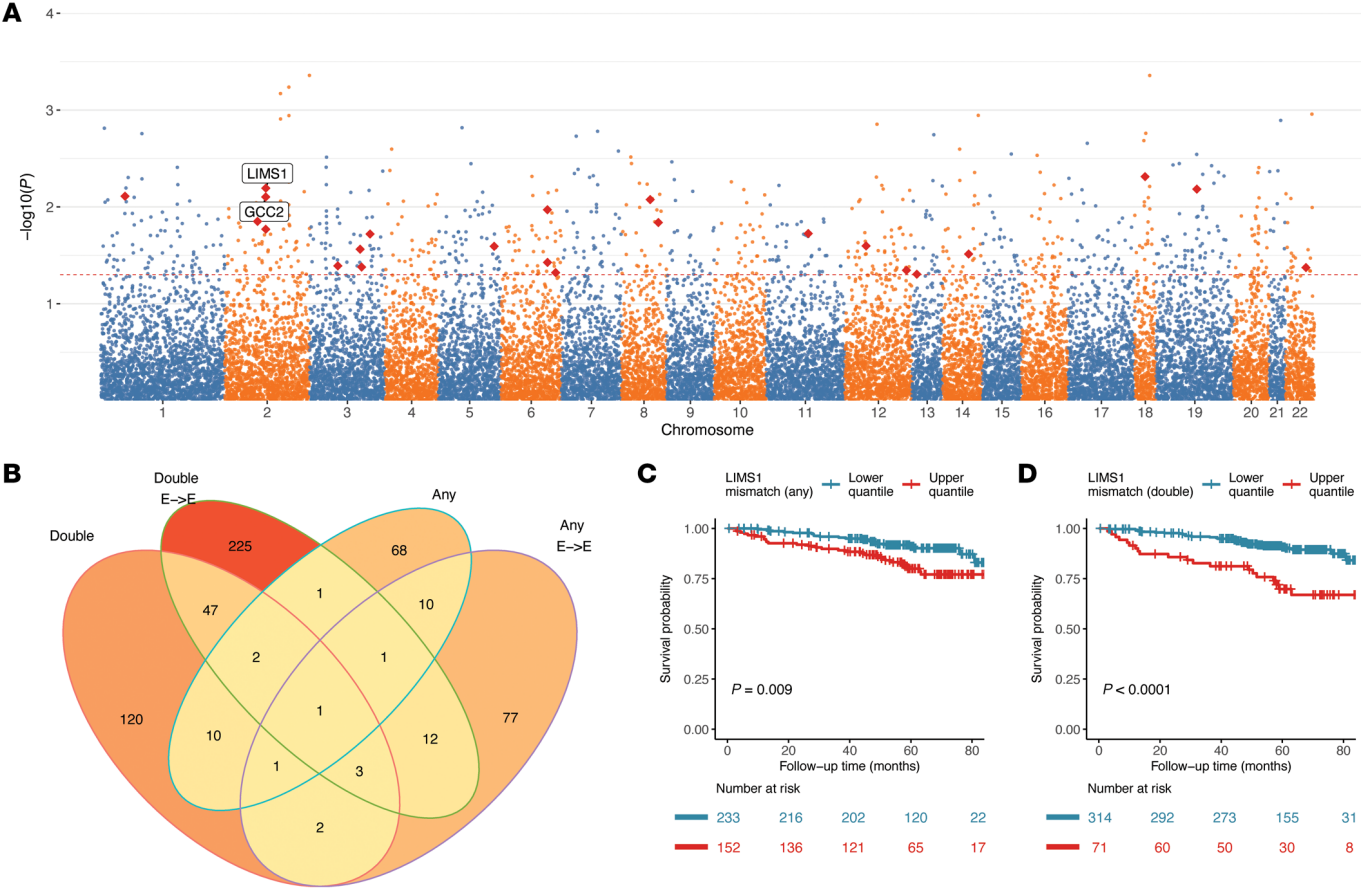
Genome-wide screening of D-R mismatch at gene level revealed the association of mismatch score at *LIMS1* locus with graft loss. (**A**) A representative Manhattan plot shows the *P* values of the association of gene-level “any mismatch” scores, with DCGL in GoCAR being 1 out of the 4 models tested (see **B** below). The 23 top candidate gene loci that were commonly identified from 4 different analyses are highlighted by red diamonds and included *GCC2* and *LIMS1*. (**B**) Venn diagram shows the number of genes identified with mismatch score at the *LIMS1* locus in significant association with DCGL (nominal *P ≤* 0.01) from 4 different analyses: double mismatch or any mismatch (definition in Figure 1 and Methods) for the whole GoCAR cohort or the subset of European-to-European (E-to-E) D-R pairs. (**C** and **D**) Kaplan-Meier plots show the graft survival curves for equally dichotomized groups of mismatch scores at the *LIMS1* locus, where mismatch scores were defined as “any mismatch” (**C**) and “double mismatch” (**D**). *P* values were derived from log-rank tests in comparison of upper quantile versus lower quantile.

We next scanned the whole genome in an effort to pinpoint specific non-HLA gene loci at which D-R mismatches are associated with graft loss. To achieve this, we derived gene-level mismatch scores by summing over variant-level mismatch scores for variants mapped to each gene region (Methods). To avoid identification of rare-variant-based mismatches while enriching for important gene-level signals, we only considered variants with relatively frequent occurrence of mismatches (≥5% D-R pairs in our study cohorts) (Supplemental Figure 1 and Methods). We then screened gene-specific mismatch scores for association with DCGL, each using a multivariable Cox regression adjusting genome-wide mismatch score, HLA mismatch score, and clinical covariates (Figure 3A). We considered any mismatch and double mismatch for all and E-to-E D-R pairs, resulting in 4 sets of analyses. Manhattan plots depicting results of these analyses are in Figure 3A and Supplemental Figure 5. In GoCAR, among 20,141 genes with derived mismatch score, we found that the gene-level mismatch score at the *LIMS1* locus was robustly associated with DCGL in all 4 scenarios, independently of genome-wide mismatch, highlighting the important role of this non-HLA locus in graft outcomes (Figure 3, B and C, Supplemental Figure 5, Supplemental Figure 6, B and C, and Supplemental Table 4). The association signal was validated in the CTOT cohort (Supplemental Table 5). Incorporation of HLA-DQ mismatches (in GoCAR) and HLA-C, DP, and DQ mismatches (in CTOT) in multivariable Cox models did not alter the relationship of *LIMS1* mismatches with DCGL (Supplemental Table 6). De novo anti-HLA donor-specific antibodies (DSAs) developed in 23 out of 385 (5.97%) GoCAR recipients, and was independently associated with DCGL (Supplemental Table 7). However, *LIMS1* mismatches were associated with DCGL when adjusted for de novo anti-HLA DSAs, suggesting this association is independent of anti-HLA DSAs (Supplemental Table 7). As sensitivity analysis, a less stringent threshold (nominal *P ≤* 0.05) led to a list of 23 candidate genes (including *LIMS1*) whose gene-level mismatch scores were associated with DCGL (Supplemental Table 4 and Supplemental Figure 6A). We summarized mismatches at the 23 gene loci into a single mismatch score by accumulating gene-wise mismatch score and evaluated its association with DCGL. In adjusted Cox models, the 23-gene mismatch score was significantly associated with DCGL, while the association of the remaining genome mismatches with DCGL was markedly attenuated, highlighting the disproportionate relevance of mismatches in these 23 non-HLA genes (Supplemental Figure 7). Notably, among the 23 genes, *GCC2* and *GCC2-AS1*, located on chromosome 2q12, are in proximity to *LIMS1*. Hence, these analyses suggested a role for mismatches in the non-HLA chromosomal region surrounding the *LIMS1* gene.

### Evaluation of variant-level mismatches in LIMS1 revealed variants associated with DCGL

**Figure 4:**
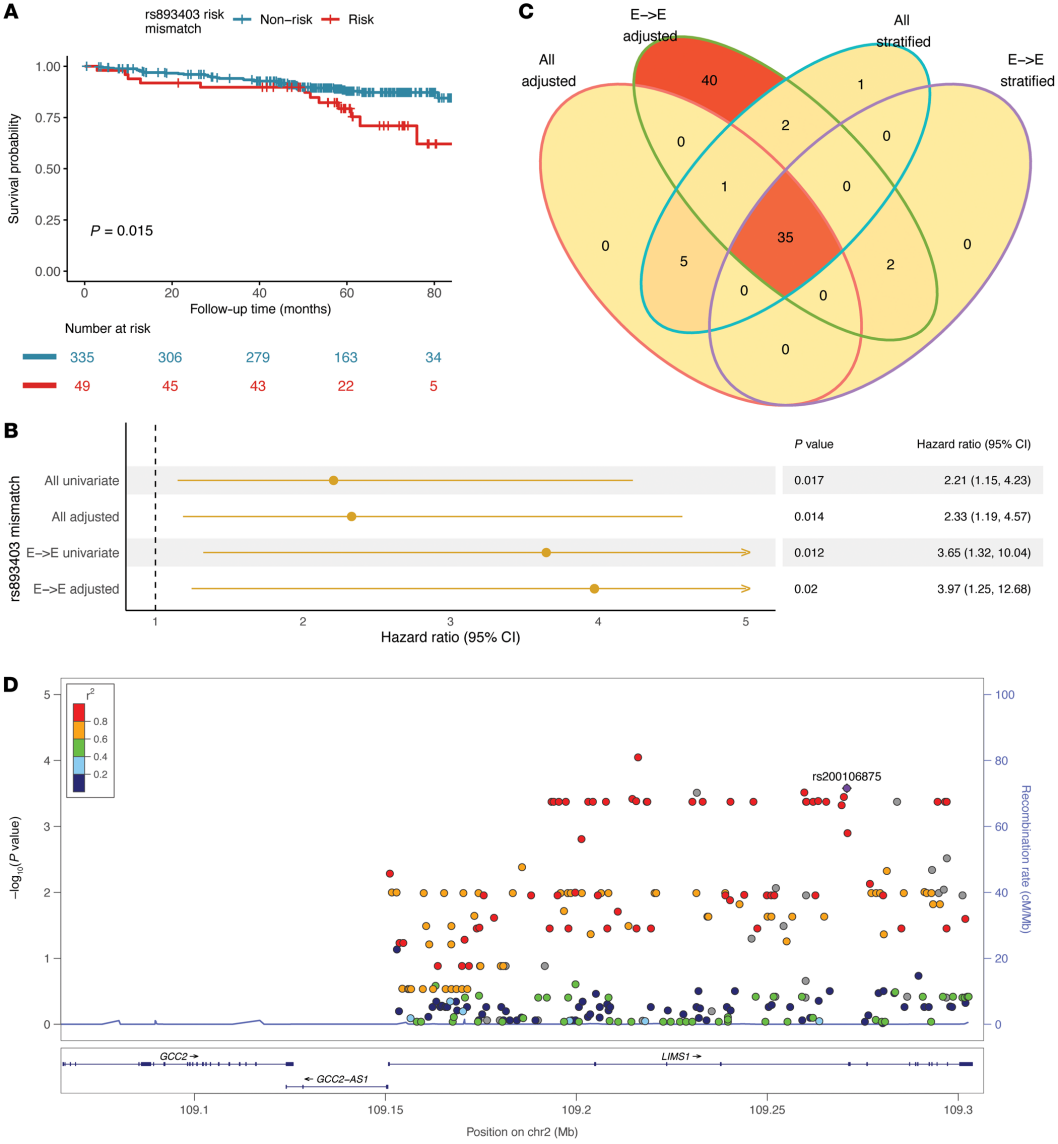
Variant-level mismatches at the *LIMS1* locus were associated with death-censored graft loss. (**A**) Kaplan-Meier plot shows that the SNP rs893403 mismatch was associated with DCGL. *P* value was derived from log-rank test in comparison of mismatch versus non-mismatch group. (**B**) Forest plot for the association of the rs893403 mismatch with DCGL in all and European-to-European (E-to-E) D-R pairs, with univariate and multivariable models adjusted by genome-wide mismatch, HLA mismatch score, donor status, and induction therapy. (**C**) Venn diagram shows the number of top candidate SNPs (other than rs893403) associated with DCGL (nominal *P ≤* 0.05) within the *LIMS1* gene region in Cox regression analyses adjusted by rs893403 mismatch or within the rs893403 non-risk stratum for all and E-to-E D-R pairs. (**D**) LocusZoom plot shows the association *P* values (on –log_10_ scale) of variants with high frequency of any mismatch (≥5% D-R pairs) within the *LIMS1* locus in adjusted analysis in all D-R pairs. The linkage disequilibrium metric *R*^2^ was calculated for SNPs surrounding 1 of the top candidate SNPs, rs200106875, within the *LIMS1* gene region.

The top candidate gene locus observed in our analyses, *LIMS1*, was also implicated in a previous study, harboring an important mismatch at intronic SNP rs893403 (9, 15) — defined by the presence of homozygosity in the CNV-tagging minor allele in the recipient and the presence of the major allele (either heterozygous or homozygous) in the donor kidney. In prior data, the rs893403 mismatch was associated with increased risk of rejection (9). In GoCAR, we first observed that the mismatch at rs893403 was associated with increased risk of DCGL in all and E-to-E D-R pairs (Figure 4, A and B).

We then evaluated variant-level D-R mismatches (any mismatch) for all non-rs893403 *LIMS1* SNPs (*n* = 287 variants after quality control; Methods) and their individual associations with DCGL. To investigate association signals independent of rs893403, we screened any mismatch of each *LIMS1* SNP using (a) Cox models adjusted by rs893403 risk mismatches (*n* = 385) and (b) stratified Cox model by excluding D-R pairs with rs893403 risk mismatches, i.e., within the rs893403 non-risk stratum (*n* = 335) (Figure 4C). The SNP-wise screening was carried out in all and E-to-E D-R pairs, resulting in 4 sets of analyses for each SNP. Supplemental Table 8 shows the top ranked (by *P* value) 30 individual SNP mismatches, which were identified as significantly associated with DCGL in all 4 scenarios. Notably, all 30 SNPs identified are located in *LIMS1* intronic regions. Using the linkage disequilibrium (LD) data derived from all the major continental populations of the 1000 Genomes Project (16), we observed that these intronic SNPs are in high LD with each other (*R*^2^ > 0.99), but are not linked with rs893403 (*R*^2^ < 0.5) (Supplemental Figure 8A), while the latter itself serves as a tagging SNP of a different haplotype within the *LIMS1* locus (Supplemental Figure 8B). Hence, we accounted for mismatches within these linked SNPs as a single block (or haplotype) distinct from rs893403 (Figure 4D and Supplemental Figure 8). In the CTOT (validation) cohort, we successfully reidentified 28 of the 30 haplotype SNPs discovered in GoCAR. Here too, D-R mismatch within this haplotype was associated with increased risk of DCGL in univariate and adjusted multivariable survival analyses (Supplemental Figure 9 and Supplemental Table 9). Comparison of allele prevalence of the *LIMS1* haplotype and rs893403 in the 1000 Genomes Project (*n* = 2504) and within GoCAR (*n* = 385 D-R pairs) revealed a 0.097-to-1 rate for the minor haplotype allele and a 0.568-to-0.98 rate for the rs893403 A allele in different ancestral populations (Supplemental Figure 10), with rare recombination within the haplotype in all ancestries (17). Notably, given the high prevalence of the minor haplotype or the A allele in East Asian populations, *LIMS1* mismatches at either locus are likely not relevant in East Asian ancestry transplants.

In summary, we confirmed and extended prior data regarding the *LIMS1* locus implicated in kidney transplant rejection, and specifically the D-R mismatch at the CNV-tagging SNP rs893403, and identified its association with DCGL. We also discovered that an independent mismatch within the *LIMS1* locus (surrogated by a 30-SNP haplotype) was associated with DCGL, overall showing the importance of this chromosomal region in allotransplantation.

### Dosage effect, directionality, and mediators of the association of D-R mismatch at the LIMS1 haplotype and graft loss

**Figure 5:**
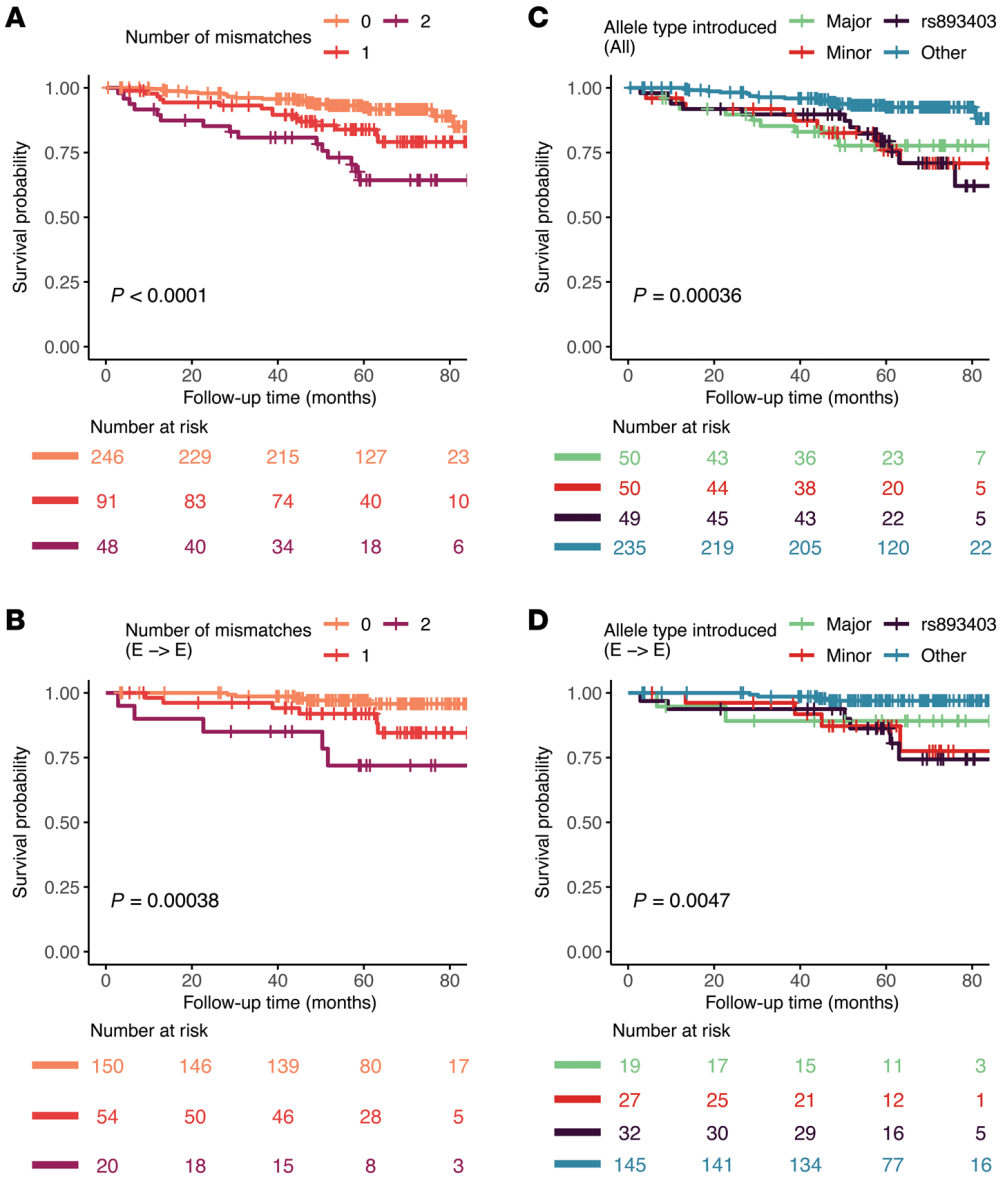
Dosage effect and directionality of the haplotype mismatch associated with DCGL. Kaplan-Meier survival curves of DCGL grouped by the dosage of mismatches (i.e., the number of “alien” alleles introduced by the donor) at the haplotype in all (**A**) and European-to-European (E-to-E) D-R pairs (**B**). Kaplan-Meier survival curves of DCGL grouped by the directionality of the mismatches at the haplotype and the rs893403 risk mismatch in all (**C**) and E-to-E D-R pairs (**D**). Major: mismatch derived from major haplotype allele introduced by donor to the recipient with homozygous minor allele and no rs893403 risk mismatch; Minor: mismatch derived from minor haplotype allele introduced by donor to the recipient with homozygous major allele and no rs893403 risk mismatch; rs893403: mismatch at rs893403 defined as risk allele (A allele) introduced by donor to the recipient carrying G/G genotype; Other: no mismatch at the haplotype and rs893403.

Using Cox regression, our analysis showed that the number of haplotype allele mismatches was associated with increased risk of DCGL in all and E-to-E D-R pairs in GoCAR, suggesting a dosage effect for D-R mismatches at this haplotype (Figure 5, A and B, and Table 1) (9). In the GoCAR cohort, we showed that the association of rs893403 mismatch with DCGL had directionality; only donor A allele introduced into recipients with a homozygous G allele (linked to the deletion) increased the risk of graft loss, consistent with existing results on the directionality of the association with acute rejection (9). We next explored the directionality of the effect on DCGL of the newly identified haplotype mismatch: donor-kidney-introduced minor allele of the haplotype to a mismatched recipient carrying a homozygous major allele, as opposed to vice versa. To minimize confounding from rs893403 mismatch, we set apart the D-R pairs with rs893403 risk mismatch into a single group (regardless the mismatch status of the haplotype). In the whole cohort, and in E-to-E D-R pairs, patients with rs893403 risk mismatch and minor haplotype allele–introducing mismatch had worse DCGL outcomes (Figure 5, C and D, and Table 2). In the E-to-E D-R pairs, the major haplotype allele–introducing mismatch was not significantly associated with DCGL. In non–E-to-E D-R pairs, both the association and directionality of haplotype mismatch with DCGL were lost (Figure 5D, Supplemental Figure 11, and Supplemental Table 10).

Overall, these analyses revealed an observable dosage effect on DCGL from *LIMS1* haplotype mismatch and suggested a directionality of this effect, i.e., worse allograft outcomes observed in D-R pairs with minor-to-major haplotype mismatch than major-to-minor mismatch, even after accounting for the rs893403 risk mismatch specifically in an ancestrally homogeneous E-to-E cohort.

To identify mediators of the association of the haplotype mismatches with DCGL, we first evaluated rejection episodes in the GoCAR cohort, as previously described (13). As shown in Supplemental Table 11, *LIMS1* haplotype mismatches were not associated with subclinical/clinical AR episodes in GoCAR. Interestingly, in ordinal logistic regression models specifically in E-to-E transplants, the number of minor *LIMS1* haplotype alleles in the donor kidney and the number of haplotype mismatches tended to associate with increased chronic allograft dysfunction index (CADI) score and interstitial fibrosis/atrophy (Ci+Ct) score obtained at 12-month surveillance biopsies (11, 18) (*n* = 151 in GoCAR; Supplemental Table 12). These suggested that *LIMS1* haplotype mismatches may associate with cumulative allograft damage.

### LIMS1 SNPs are eQTLs for GCC2 associated with Treg activation and TGF-β1/SMAD signaling

To explore the putative mechanism of the directional mismatches of the SNPs in intronic regions of *LIMS1* in contributing to DCGL, and based on the previous report of rs893403 as an eQTL in non-glomerular renal tissue (Supplemental Figure 12A) (9), we evaluated the association between *LIMS1* gene expression and the haplotype. To study the donor haplotype and gene expression in allograft kidneys, we examined the NephQTL data of published kidney transcriptomes (18). Similar to rs893403, the minor alleles of the haplotype SNPs were associated with increased expression of *LIMS1* in tubules (Supplemental Figure 12B).

**Figure 6:**
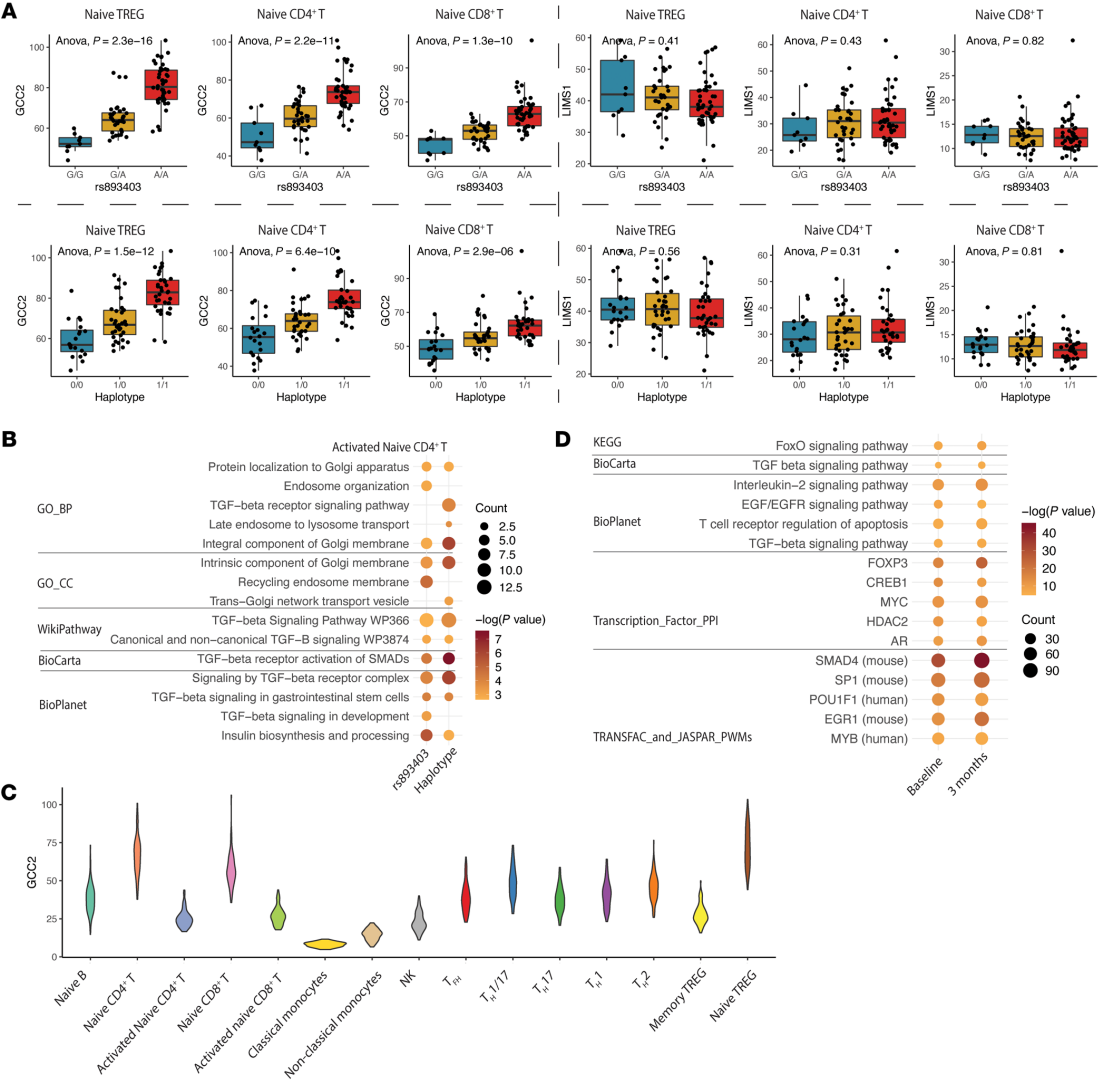
eQTL analysis of rs893403 and the haplotype revealed *GCC2* as *cis*-regulated gene. (**A**) Box-and-whisker plots show the distribution of *GCC2* and *LIMS1* expression within each genotype group of the haplotype and rs893403 in naive Tregs, naive CD4^+^ T cells, and naive CD8^+^ T cells from the DICE cohort. The significance of the association between expression level and the genotype is indicated by the *P* value derived from 1-way ANOVA. (**B**) Enriched pathways of DEGs (nominal *P ≤* 0.05) associated the number of risk alleles of rs893403 and the haplotype in naive CD4^+^ T cells from the DICE cohort. (**C**) The distribution of *GCC2* expression, displayed as violin plot, in the 15 different immune cell types from the DICE cohort. (**D**) Enriched pathways and transcription factors of genes positively coexpressed with *GCC2* (*R* ≥ 0.6, *P ≤* 0.01) in pretransplant and 3-month posttransplant patients from the GoCAR cohort. The pathways or transcription factors shown in **B** and **D** are grouped by the source databases.

To simultaneously explore the role of the haplotype alleles in the recipient during the occurrence of a “mismatch,” we evaluated the eQTL function in immune cell transcriptomes from published data (19, 20). Unexpectedly, from multiple data sets, the *LIMS1* haplotype is not an eQTL for *LIMS1* mRNA in peripheral blood mononuclear cells (PBMCs). Instead, both the *LIMS1* haplotype and rs893403 demonstrated significant eQTL function on *GCC2*, the 5′-end-neighboring gene to *LIMS1* (Figure 6A and Supplemental Figure 13). *GCC2* (or Golgin-185) is a GRIP domain–containing protein with a canonical role in late endosome–to-Golgi transport by binding Rab and Arl1 GTPases (21–23) and is essential for mannose-6-phosphate receptor (M6PR) trafficking (23). In turn, cation-independent M6PR (CI-M6PR or *IGF2R*) has roles in binding and activation of latent TGF-β1 by cleavage of latent peptide (promoting TGF-β1 signaling) (24, 25), and in insulin-like growth factor-2 (*IGF2*) signaling (25–27).

Using the published DICE (Database of Immune Cell Expression, Expression quantitative trait loci and Epigenomics) cohort (19), where sorted PBMCs after leukapheresis from healthy controls were genotyped and underwent RNA-seq (Methods), we successfully re-identified 22 of the 30 haplotype SNPs, which also showed consistent genotypes across samples. From these data of sorted immune cell populations, Supplemental Table 13 shows the rank of *GCC2* among genes across the transcriptome (by Benjamini-Hochberg–adjusted *P* value) in the eQTL analysis of rs893403 and the haplotype in each of the 15 sorted PBMC subtypes, among which naive Tregs were top ranked (Methods). We also evaluated transcriptome-wide differentially expressed genes (DEGs) based on rs893403 and the *LIMS1* haplotype genotype (Methods). Consistent with the described role of GCC2, we identified enrichment of endosomal transport, TGF-β1/SMAD signaling, and IGF2 signaling in these analyses (Figure 6B and Supplemental Figure 14).

We next studied *GCC2* expression levels in immune cell subsets of the DICE data. *GCC2* was highly expressed in naive Tregs, naive CD4^+^ and CD8^+^ T cells, and was downregulated upon T cell receptor stimulation (Figure 6C). *LIMS1* mRNA expression was relatively high in monocytes (Supplemental Figure 15A). Relative expression of *GCC2* and *LIMS1* in PBMC subsets was corroborated in our own PBMC single-cell sequencing data obtained from patients with end-stage kidney disease (Supplemental Figure 15B) (13).

To explore the function of *GCC2* in peripheral blood cells using data from the parent GoCAR study, we performed coexpression analyses using previously obtained whole-blood transcriptomes from recipients, both before transplantation (GSE112927) and at 3 months after transplantation (GSE120398) (*n* = 292 and 147, respectively). As shown in Figure 6D, *GCC2*-coexpressed genes at both time points (*R* ≥ 0.6; adjusted *P ≤* 0.05) (*n* = 571 and 853 genes, respectively) showed significant and consistent enrichment of TGF-β1/SMAD signaling pathway terms and FOXP3 transcription factor targets throughout multiple databases (also see Methods). *GCC2*-coexpressed genes (*R* ≥ 0.6; *P ≤* 0.05) in each cell type from the healthy controls of the DICE cohort also showed enrichment of TGF-β1/SMAD signaling pathway terms and *FOXP3* in multiple T cell subsets (Supplemental Figure 16 and Methods).

Our analysis here implies a regulatory role for rs893403 and the *LIMS1* haplotype in *GCC2* expression in immune cell subsets, including Tregs, and further links *GCC2* expression with the described canonical function relating to TGF-β1/SMAD signaling in immune cells.

### Prioritizing candidate SNPs at the LIMS1-GCC2 locus with functional genomics annotation

**Figure 7:**
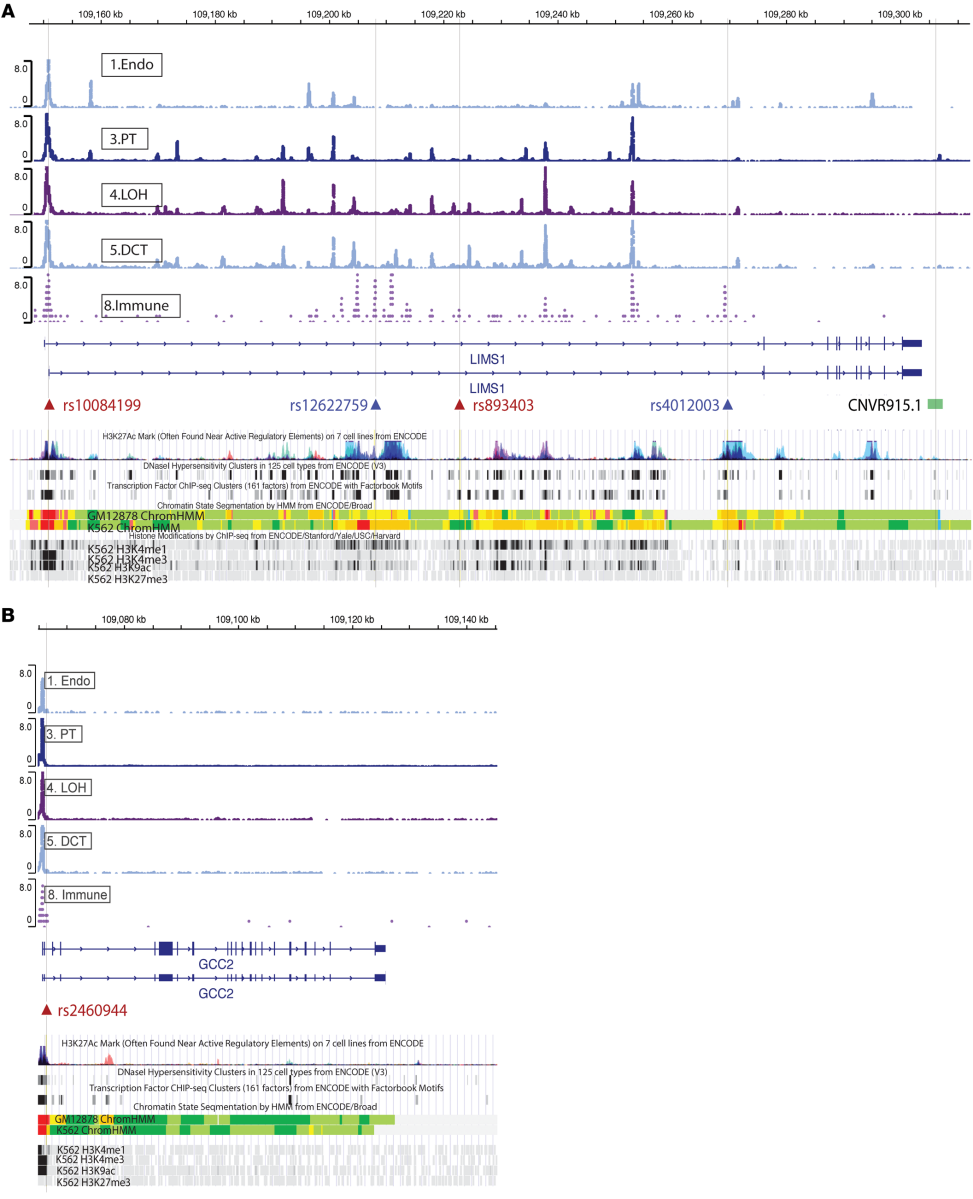
Potential regulatory SNPs in high LD with rs893403 and the haplotype in the *LIMS1* and *GCC2* gene regions. Cell-specific peaks from whole-kidney single-cell ATAC-seq data (28) are shown for *LIMS1* (**A**) and *GCC2* (**B**). SNP IDs in red are in LD with rs893403, while SNP IDs in blue are in LD with the haplotype. Annotation of epigenetic and transcription factor ChIP-seq data in corresponding tracks is from the UCSC genome browser. The deletion (CNVR915.1) tagged by rs893403 (9) is denoted as a green bar.

Since our above analyses suggested eQTL functions of rs893403 and the *LIMS1* haplotype for *GCC2*, we aimed to further prioritize these SNPs by integrating publicly available data sets, including assay for transposase-accessible chromatin with high-throughput sequencing (ATAC-seq) data (28), ChIP-seq data (29), DNase hypersensitivity (30), transcription factor binding (30), and ENCODE data. Our goal was to identify putative “causal” SNPs in our list of candidate *LIMS1* SNPs, including rs893403. As shown in Figure 7, rs893403 is located in an ATAC-seq peak region specific to immune cells, while there are no peaks detected within the previously described CNV (CNVR915.1) tagged by rs893403. Evaluation of SNPs highly linked (*R*^2^ > 0.9) to rs893403 revealed 2 candidate SNPs located in accessible chromatin areas, rs2460944 (*R*^2^ = 0.96) within the transcription start site (TSS) of *GCC2* and rs10084199 (*R*^2^ = 0.91) in the TSS of *LIMS1*. These SNPs showed DNase hypersensitivity, high RegulomeDB scores (Methods) (31), transcription-favorable histone modifications, and confirmed highly significant eQTL function in multiple tissues for *GCC2* based on the genotype-tissue expression (GTEx) project data (20) (Supplemental Tables 14 and 15). SNP rs2460944 mismatches were found to be associated with DCGL, with similar directionality as rs893403 (Supplemental Figure 17).

Among candidate SNPs within the *LIMS1* haplotype, rs4012003 and an additional SNP, rs12622759, are located in areas of open chromatin, with further evidence of DNase hypersensitivity, high RegulomeDB scores, favorable histone modifications, and significant eQTL function for *GCC2* in multiple cell types. These peak regions appear specific to immune cells according to the scATAC-seq data. These in silico analyses suggest putative “causal” SNPs within the *LIMS1-GCC2* locus, which are tagged by rs893403 or within the *LIMS1* haplotype and could regulate *GCC2* expression.

### GCC2 modulates active TGF-β1 levels and SMAD signaling in lymphocytes and epithelial cell lines

**Figure 8:**
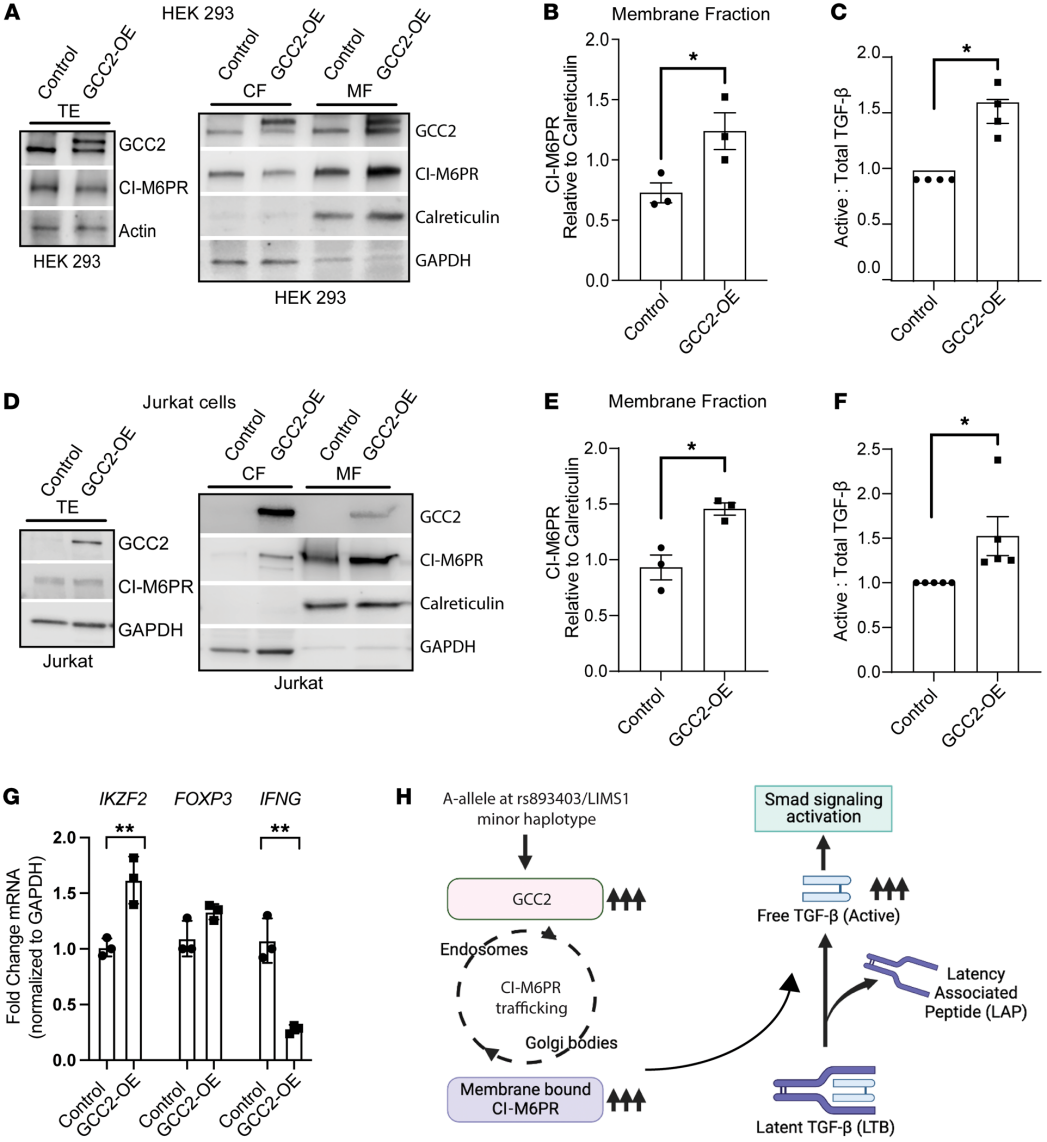
*GCC2* modulates generation of active TGF-β1 and downstream signaling in lymphocytes and epithelial cell lines. To investigate the cellular role of *GCC2*, we overexpressed either a GFP-tagged GCC2 (GCC2-OE) or a GFP-control expression plasmid in HEK-293T cells (**A**–**C**) and Jurkat T cells (**D**–**F**), followed by extraction of protein lysates, subcellular fractionation, and immunoblotting. Representative immunoblots of cellular fractions probed for GCC2, CI-M6PR, calreticulin, and GAPDH are displayed for HEK-293T (**A**) and Jurkat T cells (**D**). Dot plots show corresponding densitometric quantifications of CI-M6PR in the MFs (normalized to calreticulin) from these respective cell lines (**B** and **E**). Dot plots show corresponding ratios of active (LAP cleaved) to total TGF-β1 levels (both in pg/mL normalized to control in each paired set and analyzed by paired, 2-tailed *t* test) in GCC2-OE and controls in HEK-293T (**C**) and Jurkat T cells (**F**) supernatants assayed by ELISA after 24 hours serum starvation (*n* ≥ 4 sets). (**G**) Dot plots show qPCR results for *IKZF2*, *FOXP3*, and *IFNG* mRNA normalized to *GAPDH* in GCC2-OE and control Jurkat T cells (*n* = 3 sets). (**H**) Schema showing role of *LIMS1* locus mismatch (A allele at rs893403) in activation of SMAD signaling pathway in response to increased *GCC2* levels. GCC2 overexpression leads to increased levels of membrane-bound CI-M6PR levels via Golgi-to-endosome trafficking causing higher levels of active TGF-β1 and ultimately SMAD pathway activation (figure created using BioRender). *P < 0.05, **P < 0.01 by 2-tailed, unpaired *t* test. MF and CF, membrane and cytoplasmic fraction of lysate; TE, total extract; CI-M6PR, cation-independent mannose-6-phosphate receptor; IMCD, rat inner medullary collecting duct cells; TGF-β, TGF-β1.

Our in silico data are consistent with the reported roles of *GCC2* (or Golgin-185) in the trafficking of, and cell-surface abundance of, CI-M6PR, followed by activation of latent TGF-β1 by the cleavage of latency-associated peptide (LAP) (32), promoting autocrine or paracrine TGF-β1 signaling in T cells and/or in epithelial cells. Based on this, we evaluated in vitro the generic role of *GCC2* in the trafficking of and membrane abundance of CI-M6PR and in regulating TGF-β1/SMAD signaling. We first overexpressed a GFP-tagged *GCC2* construct (or GFP-control) in HEK-293T cells and Jurkat T cells and evaluated candidate proteins involved in this signaling axis (Figure 8, A–F). Overexpression of GFP-GCC2 significantly increased the abundance of membrane-associated CI-M6PR versus GFP-controls (Figure 8, A, B, D, and E). In both of these cell types, GCC2 overexpression led to increased ratios of LAP-cleaved, active (free) TGF-β1 to total TGF-β1 levels in the supernatant (Figure 8, C and F). Similar findings of increased CI-M6PR and active-to-total TGF-β1 levels were also identifiable in inner medullary collecting duct (IMCD) cell lines (Supplemental Figure 18, A–C). In Jurkat T cells, GCC2 overexpression increased transcripts of *IKZF2* (Helios) (33), tended to increase *FOXP3*, and reduced *IFNG* (34, 35) (Figure 8G). We then used lentiviral shRNA clones to knock down *GCC2* and observed significantly reduced phospho-SMAD3 downstream of TGF-β1 in HEK-293T cells versus a scrambled shRNA (Supplemental Figure 18D). Total CI-M6PR was not significantly altered either by GCC2 overexpression or knockdown, suggesting that the effect should be mediated indirectly, via altering protein trafficking (Figure 8, A and D, and Supplemental Figure 18D). Analogously, in both Jurkat T cells and IMCD cells, GCC2 knockdown tended to reduce active-to-total TGF-β1 levels in the supernatant (Supplemental Figure 18, E and F). These experiments connect the modulation of cellular *GCC2* levels by the *LIMS1* haplotype or rs893403 with the generic role of GCC2 in M6PR trafficking and in regulating the TGF-β1 signaling pathway in multiple cell types, including specifically in T cells (schema in Figure 8H).

## Discussion

The traditional paradigm regarding mismatches between donor and recipient is the development of an adaptive immune response against donor proteins of dissimilar peptide sequence by the recipient’s allorecognition mechanisms. These responses are primarily determined by mismatches between the HLA regions of donor and recipient (5–8). Here, a systematic approach screening the whole genome to discover specific non-HLA gene loci revealed *LIMS1* D-R mismatches as a top-ranked candidate associated with DCGL. Within the *LIMS1* locus, the role and directionality of mismatches at the previously reported intronic SNP rs893403 was extended to long-term DCGL. In addition to rs893403, a haplotype of 30 intronic *LIMS1* SNPs, almost perfectly linked with each other, was identified where mismatches were independently associated with increased risk of DCGL, again with a demonstrable directionality (minor allele in donors introduced into major allele–carrying recipients). Hence, our analyses provide a pipeline for identifying the role of non-HLA gene mismatches relevant to allograft survival.

Neither rs893403 nor the SNPs in the *LIMS1* haplotype alter the LIMS1 protein sequence; instead, both were identified as *cis*-eQTLs for an adjacent gene, *GCC2*, in immune cells, with high expression in Tregs. Our subsequent transcriptomic analyses revealed the association of *LIMS1* SNPs with TGF-β1/SMAD signaling via *GCC2* expression in multiple T cell subsets — including Tregs and naive CD4^+^ and CD8^+^ T cells. These transcriptomic data are consistent with the canonical role of *GCC2* (or Golgin-185) as a *trans*-Golgi network protein with a central role in the Golgi-to-endosome trafficking of M6PRs. Our experimental data (Figure 8 and Supplemental Figure 14) using multiple cell lines confirmed the effects of GCC2 overexpression (i.e., analogous to the increased expression induced by an A allele at rs893403 or minor allele at the *LIMS1* haplotype) on the abundance of membrane-bound CI-M6PR. In turn, changes in surface CI-M6PR levels with GCC2 overexpression/knockdown modulated active TGF-β1 levels and SMAD signaling. In Jurkat T cells, we demonstrated that GCC2 overexpression increased Helios expression (associated with Treg stability and function) and reduced proinflammatory *IFNG*, in line with in silico and clinical data.

While we validated and extended previous association of the *LIMS1* rs893403 mismatch to allograft survival, we could not find significant associations with either antibody-mediated acute rejection (ABMR) (9) or T cell–mediated rejection (TCMR) (15) in our data sets. We note that our study cohorts reported subclinical and clinical rejection episodes up to 2 years by respective central pathology cores, but later episodes were not required to be reported in these data sets. Indeed, we identified an association of the minor allele of the intronic *LIMS1* SNP haplotype, or alternatively the A allele at rs893403 (accompanying a high-risk mismatch), with allograft fibrosis score at 12 months (CADI or Ci+Ct in GoCAR), which we previously reported as associated with DCGL (11). These data are consistent with the previously reported role for *LIMS1* in fibrosis via facilitating ILK signaling and as markers of incomplete epithelial-mesenchymal transition in tubular cells, another potential mechanism linking donor minor haplotype to adverse allograft outcomes (36). In addition, these data raise the possibility of a role for *GCC2* itself in renal epithelial cells, with minor-haplotype allografts associated with increased IF/TA via TGF-β1 signaling (37, 38), which we and others have reported as associated with early allograft fibrosis by gene expression profiling. On the other hand, the deletion-tagging allele (G allele) in recipients with a high-risk mismatch (i.e., A allele donors to G/G recipients) (9) was associated with lower *GCC2* levels in T cells and likely affected Treg function, via reduced TGF-β1/SMAD signaling. A recent report identified increased autoimmunity in global GCC2^–/–^ (knockout) mice with development of autoantibodies against a wide range of antigens (34). In line with these observations, the prior reported detection of anti-LIMS1 antibodies in recipients with a high-risk mismatch could itself reflect impairment of Treg function in G/G recipients, and autoantibody development. Hence intronic regulatory loci in the context of a “directional” *LIMS1* mismatch exert cell-specific effects by modulating gene expression, in turn affecting allograft survival. Our work sheds light on *LIMS1* locus mismatches and allograft outcomes by identifying these intronic SNPs as eQTLs for *GCC2* in T cells, impinging on its expected role in TGF-β1 signaling via modulating CI-M6PR levels, thus revealing an unexpected mechanism outside of the usual paradigm involving non-HLA D-R mismatches.

Our data have potential clinical implications. First, given the high allelic prevalence of minor alleles in most populations (~0.367–0.622, except in African and East Asian), the occurrence of a D-R mismatch at these loci is expected to be a common occurrence in the clinical context. Next, both the occurrence of a haplotype mismatch and the directional association with DCGL were best observed in ancestrally homogeneous E-to-E D-R pairs in our cohort. In combination with prior reports (9, 15), our data highlight the important role of *LIMS1* locus mismatches and this non-HLA genomic region in renal allotransplantation. Genotyping for these variants to delineate a high-risk mismatch could potentially allow for risk stratification — for personalized optimization of immunosuppression, or early surveillance for IF/TA or rejection (based on the prior work), or for enriching patient enrollment in subsequent clinical trials based on *LIMS1* mismatches. Given the current organ shortage, we believe *LIMS1* mismatches are not likely to be utilized for organ allocation, except in exceptional circumstances (for instance, where 2 living donors are considered for the same recipient). Nonetheless, our identification of the role of *LIMS1*, *GCC2*, and other non-HLA candidates paves the way for further detailed mechanistic studies with the potential for subsequent targeted therapeutics in case of an identified mismatch.

While the independent association of *LIMS1* gene–level mismatches with DCGL was validated across both cohorts, intergenic regions were not evaluated in our approach — i.e., only loci within annotated gene boundaries were considered. By integrating regulatory data from public data sets, we identified candidate SNPs in high LD with rs893403 or the *LIMS1* haplotype, which could potentially represent causal *cis*-eQTLs for GCC2 (Figure 7). For instance, rs2460944 is highly linked (*R*^2^ = 0.96) with rs893403, has high RegulomeDB scores (31), and is located within the transcription start site of *GCC2*. However, this and other candidates identified here will need experimental validation in future studies by altering these linked loci individually and evaluating GCC2 expression and clinical outcomes. While the association of rs893403 mismatches with rejection (9) and the association of genome-wide mismatches with DCGL (5) were also previously identified in independent cohorts, we acknowledge that the association of these 2 variables with DCGL was not confirmed in our validation cohort in adjusted analyses. We also acknowledge that we did not test for anti-LIMS1 antibodies in recipient sera, identified in recipients of high-risk mismatches in the prior report, which currently has no commercial assay. Finally, while in silico data suggested a role of SNP-mediated *GCC2* expression in T cell TGF-β1 signaling, we did not directly evaluate SNP-associated alterations in T cell subsets or *TGFB1* signals, and further mechanistic work is needed to quantitatively evaluate specific T cell subset functional changes vis-a-vis rs893403 or the *LIMS1* haplotype.

In summary, using 2 prospective kidney transplant cohorts, we identify a key role for D-R mismatches at the *LIMS1* locus using a systematic screening approach of D-R mismatches at multiple genomic scales. Furthermore, our data reveal that these intronic variants at the *LIMS1* locus have *cis*-regulatory function and D-R mismatches generated at these variants impact allograft outcomes without altering protein sequences. The mechanisms described here provide insight into our understanding of D-R mismatches and are informative in ongoing efforts to improve long-term graft survival.

## Methods

### Genotyping and imputation for GoCAR and CTOT-01/17.

The genotyping, quality control, and imputation for the GoCAR and CTOT cohorts have been described in our previous study (22, 37). Briefly, donor DNA was obtained from either pre-perfusion allograft biopsies (in deceased donors) or PBMCs (in living donors), while recipient DNA was obtained from PBMCs. The extracted DNA was genotyped with Illumina Human OmniExpressExome Array for GoCAR and Illumina Infinium Global Screening Array for CTOT. Samples with (a) genetically inferred sex not matched with reported sex; (b) missing-genotype rate greater than 0.03; and (c) excessive genome-wide heterozygosity, an indication of sample contamination were excluded. SNPs with (a) missing rate greater than 0.05; (b) minor allele frequency less than 0.01; and (c) Hardy-Weinberg equilibrium *P* value of less than 1 × 10^–6^ were excluded.

We performed genome-wide genotype imputation on both cohorts. For GoCAR, the imputation analysis was done by the pipeline composed of SHAPIT (39) and IMPUTE2 (40) software packages using the 1000 Genomes Project phase I data (41) as the reference panel, and for CTOT, the imputation was done by the Michigan Imputation Server (https://imputation-server.sph.umich.edu) (42) using the Haplotype Reference Consortium reference panel (release 1.1) (43). After imputation, an imputed genotype with a posterior probability of less than 0.95 was set as missing data.

As shown in Supplemental Figure 1, quality control on the imputed genome-wide genotype data of GoCAR and CTOT was performed separately in each cohort following the same strategy. The imputed SNPs with low confidence (INFO score < 0.4, GoCAR; *R*^2^ < 0.3, CTOT) or missing rate of 5% or less were considered low quality and excluded. For instance, the rs893403 genotype was not confidently imputed in CTOT, resulting in 52 out of 146 (35.6%) missing values in the D-R mismatch calculation, and thus was not used for validation. SNPs with no alternative alleles across the samples (i.e., monomorphic) or within the MHC region (chr6: 28866528–33775446 [hg19]) were excluded from downstream analysis as well. SNPs with mismatch carried by 5% or more of the D-R pairs were considered less frequent and removed from gene- and variant-level screening analyses.

Variants were annotated in terms of genomic locations (exonic, intronic, intergenic, etc.) or protein-coding functions (synonymous, nonsynonymous, frameshift, etc.) with ANNOVAR (version 2018Apr16) (44) with Refseq hg19 assembly. The transmembrane or secreted genes were defined with the key words from Uniprot as described in a former study (5): “transmembrane [KW-0812]” OR annotation: (type:transmem) OR locations: (location: “secreted [SL-0243]”) AND organism: “*Homo* *sapiens* (human) [9606].”

### Definition of D-R mismatch at different genomic scales.

The D-R mismatch for each variant (SNP and small indel) was defined following the strategy in a former study (5). A mismatch was defined as a donor carrying an allele that was not presented in the recipient. We consider mismatch derived from 1 “alien” allele introduced by donor as “single mismatch,” while 2 alleles introduced as “double mismatch.” The “single mismatch” and “double mismatch” were collapsed into a class named “any mismatch” (Figure 1). The mismatch score was calculated for each variant among each D-R pair. To define gene-level mismatch score, the mismatch status (0 for absence and 1 for presence) of all the variants within each annotated gene region were summed up. Similarly, to define mismatch score at a different genomic scale, such as genome-wide, exonic, non-exonic, or all transmembrane or secreted gene regions, the mismatch status of all the variants within the corresponding genomic regions were summed as the raw score and further normalized by the IQR of the corresponding raw scores across D-R pairs.

### eQTL and DEG analysis with DICE data.

To explore the gene expression regulation effect of rs893403 and the identified haplotype in immune cells, we utilized the RNA-seq and genotype data generated from the DICE project (https://dice-database.org/). Request (request number: 97206-2) for the access to the DICE data deposited in the Genotypes and Phenotypes (dbGaP) database (https://www.ncbi.nlm.nih.gov/gap/; accession number: phs001703) was approved. The detailed description of the data set can be found in the original paper (19). Briefly, whole-transcriptome bulk RNA-seq was performed on 15 immune cell types isolated from leukapheresis samples of 91 healthy donors. Gene expression was measured as transcripts per million (TPM). The raw TPM expression profile was then log_2_-transformed by log_2_(TPM + 1). Genome-wide genotype data were generated by Illumina Infinium Multi-Ethnic Global-8 array, followed by imputation with the same pipeline as we applied to the CTOT cohort (see above). The number of risk alleles (A allele) of rs893403 and the number of minor alleles of the haplotype were counted in each sample. The association of expression of each gene with the genotype of rs893403 or the haplotype was tested with limma (45), using an additive model of the number of risk alleles adjusted by age, sex, and race. Genes with a nominal *P* value of 0.05 or less were identified as DEGs. Gene set enrichment analysis of DEGs was performed with R package enrichR (46), and gene sets with enrichment nominal *P* value of 0.05 or less were considered significant.

### Coexpression and functional enrichment analysis for GoCAR PBMC transcriptomes.

The details of the RNA-seq experiment and analysis of the PBMCs of a subgroup of GoCAR patients before transplant and 3 months after transplant were described in our published studies (14, 47). The normalized data were downloaded from the NCBI Gene Expression Omnibus (GEO) database (accession numbers GSE112927 and GSE120398). Gene coexpression was evaluated by Pearson’s correlation between the expression values of each gene pair for pretransplant and 3-month posttransplant data sets separately. Genes with Benjamini-Hochberg–adjusted *P* value of 0.05 or less and absolute correlation coefficient |*R*| ≥ 0.6 were considered coexpressed, followed by gene set enrichment analysis as described above.

### Online tools for in silico analyses.

The LD matrix (on GRCh37) for haplotype SNPs and rs893403 was generated with LDmatrix from the LDlink online portal (https://ldlink.nci.nih.gov/?tab=home) using all major continental populations from the 1000 Genomics Project as reference. SNPs in LD with rs893403 and the haplotype were identified by LDproxy from the LDlink online portal using the same reference. SNPs with *R*^2^ greater than 0.9 were considered in high LD with targeted SNPs, and each SNP was annotated with the Regulomedb database (29) through LDproxy. The Regulomedb score was defined based on the strength of the supporting data as described in https://regulomedb.org/regulome-help/, with a lower value indicating stronger confidence of the regulatory role of the corresponding SNP. The SNPs were mapped to human kidney scATAC-seq peaks using the Susztaklab Kidney Biobank (28) with human genome hg19 assembly. The H3K27Ac mark, DNase I hypersensitivity clusters, transcription factor ChIP-seq clusters, chromatin state segmentation, and histone modification information were generated from the UCSC Genome Browser with human genome hg19 assembly (48). The eQTL analysis box-and-whisker plots for *LIMS1* gene expression associated with rs893403 and the haplotype genotype in kidney tubulointerstitial tissue were generated from the NephQTL online portal (https://nephqtl.org) (18). The LD structure between SNPs (on genome build hg19) within the *LIMS1* gene region and the identified haplotype (represented by rs200106875) was evaluated by locuszoom/1.4 (49), with major continental populations from the 1000 Genomes Project as reference.

### Cell lines.

Jurkat T cells were obtained from ATCC and grown in complete RPMI 1640 medium supplemented with 10% fetal calf serum. For Jurkat cell activation/proliferation, anti-CD3 (OKT3 FG; 16-0037-85, Thermo Fisher Scientific) at 1 μg/mL and anti-CD28 cocktail (16-0289-85, Thermo Fisher Scientific) at 3 μg/mL were applied. HEK-293T (ATCC) and IMCD (gift from Stefan Somlo, Department of Medicine, Yale University School of Medicine) kidney tubular cell lines were grown in complete DMEM and F12/DMEM (GIBCO), respectively, supplemented with 10% fetal calf serum. At indicated times in each experiment (48–72 hours after transfection/electroporation), cells were centrifuged at 500*g* and 4°C for 5 minutes.

### In vitro GCC2 overexpression studies.

A human *GCC2* construct (encoding full-length GCC2 C-terminus from Open Biosystems) was cloned into mammalian expression vector pcDNA3.1-C-eGFP with Neomycin resistance gene (Genscript) with a C-terminal GFP tag. The GCC2-GFP construct or GFP-control was overexpressed in HEK-293T cells using TurboFect (Thermo Fisher Scientific), or via electroporation into IMCD (125 V, 5 ms, Nepagene) and Jurkat T cell lines (300 V, 1 ms, Nepagene) using the NEPA21 electroporator as per the manufacturer protocols. For expression of full-length GCC2 fluorescent fusion proteins, 10 μg plasmid in 100 μL Opti-MEM was used to electroporate 1 × 10^6^ Jurkat T cells or IMCD cells. Forty-eight to 72 hours after transfection, expression was verified by fluorescence microscopy. G-418 was used to select and enrich for expressing clones.

### In vitro GCC2-knockdown studies.

Lentiviral human *GCC2* constructs (short hairpin clones; Dharmacon, Inc.) were tested for optimal suppression of *GCC2* expression in HEK-293T cells, to generate stable GCC2 knockdown in HEK-293T, IMCD, and Jurkat T cells (gift from Dan Jane-Wit, Department of Medicine, Yale University School of Medicine). The selected RFP-tagged (red fluorescent protein) hairpins and respective scrambles were used to generate a mammalian VSV pseudotyped lentiviral expression construct. Lentiviral medium was used to infect each of the human HEK-293T, IMCD, and Jurkat T cell types and were grown in puromycin-contining (1 μg/mL) DMEM/F12 medium or RPMI 1640, respectively, for experiments after at least 7 days of selection at 37°C.

### Total protein extraction.

To obtain total cell extracts, cells were lysed with a buffer containing 25 mM Tris-HCl pH 7.4, 150 mM NaCl, 1 mM EDTA, 1% NP-40, and 5% glycerol, a protease inhibitor mixture, and tyrosine and serine/threonine phosphorylation and phosphatase inhibitors. Extracts were quantified using BCA protein estimation and denatured using Laemmli SDS sample buffer.

### Subcellular fractionation.

Hypotonic lysis and fractionation were performed using a Pierce Subcellular Protein Fractionation Kit (Thermo Fisher Scientific, 78840, 35 mL). Cytoplasmic and membrane extracts were prepared according to the manufacturer’s protocol. Briefly, the cells were resuspended in cytoplasmic extraction buffer with protease inhibitors for 10 minutes at 4°C on a rocker. Cytoplasmic extracts were obtained by spinning down at 500*g* for 5 minutes, followed by resuspending in membrane extraction buffer with protease inhibitors for 10 minutes at 4°C on a rocker. Membrane extracts were obtained by spinning down at 3,000*g* for 5 minutes. Extracts were quantified using BCA protein estimation and denatured using Laemmli SDS sample buffer.

### Western blotting.

Overexpression of GCC2 was confirmed by immunoblotting for each of the cell lines used. For SMAD3 phosphorylation and TGF-β1 supernatant studies, overexpressing cell lines were serum starved overnight. Total and fractionated extracts were analyzed by immunoblot using a rabbit anti-GCC2 polyclonal antibody (PA5-89457, Invitrogen), anti–IGF-II receptor/CI-M6PR (D3V8C) rabbit monoclonal antibody (14364, Cell Signaling Technology [CST]), anti–phospho-SMAD3 rabbit monoclonal antibody (pS423/425) (C25A9) (9520, CST), anti-SMAD3 rabbit monoclonal antibody (9523, CST), rabbit calreticulin antibody supernatant (CPTC-CALR-1-s, DSHB), anti-GAPDH rabbit monoclonal antibody (14C10) (2118, CST), and anti–β-actin mouse monoclonal antibody (A5441, Sigma-Aldrich). Densitometry was performed on images of Western blots using ImageJ software (NIH).

### TGF-β1 ELISA.

The supernatants were collected and centrifuged at 500*g* and 4°C for 5 minutes, and the supernatants were collected carefully and stored at –20°C prior to ELISA. TGF-β1 activity was estimated by ELISA using a LEGEND MAX Free Active TGFβ-1 ELISA Kit and LEGEND MAX Total TGF-β1 ELISA Kit (BioLegend) according to the manufacturer’s protocols. Briefly, standards and 50 μL of supernatants were loaded into the wells, and the absorbance were measured at 450 nm using a Synergy-LX multimode reader (BioTek) and analyzed by Gen5 Microplate Reader and Imager software (BioTek). Each of the standard and samples were tested in duplicate.

### Reverse transcription and quantitative PCR.

RNA was extracted using an RNA extraction kit (Thermo Fisher Scientific GeneJET RNA Purification Kit) and was transcribed into cDNA using a cDNA Reverse Transcription Kit (Thermo Fisher Scientific Maxima First Strand cDNA Synthesis Kit for RT-qPCR), with starting total RNA at approximately 1,000 ng. Gene expression at the transcript level was assayed in vitro by real-time PCR using SYBR Green reagents and an automated 7500 software platform (Applied Biosystems StepOne Plus Real-Time PCR systems, Thermo Fisher Scientific).

Specific primers were designed for the following human genes: *IFNG* (Forward [Fw] primer: TCGGTAACTGACTTGAATGTCCA and Reverse [Rv] primer: TCGCTTCCCTGTTTTAGCTGC), *IKZF2* or Helios (Fw primer: TCACCCGAAAGGGAGCACT and Rv Primer: CATGGCCCCTGAT-CTCATCTT), *FOXP3* (Fw primer: GTGGCCCGGATGTGAGAAG and Rv primer: GGAGCCCTT-GTCGGATGATG). Amplification curves were analyzed via the 2^–ΔΔCt^ method. *GAPDH* (Fw primer: GGAGCGAGATCCCTCAAAAT and Rv primer: GGCTGTTGTCATACTTCTCA-TGG) was used as endogenous control.

### Statistics.

The associations of time-to-event outcomes (e.g., DCGL) with risk factors (e.g., D-R mismatches) were evaluated by Cox regression implemented in R package survival, with other relevant factors adjusted as covariates. Samples with missing data in covariates were omitted. For categorical (e.g., rejection episodes) or ordinal outcomes (e.g., CADI-, Ci-, Ct-, i-, t-scores at 12 months after transplant), logistic or ordinal regressions were performed to investigate the association with risk factors, respectively. For the box-and-whisker plots, the line in the middle shows the median value, while the lower and upper regions show the 25th and 75th percentiles. The upper whisker extends to the largest value no more than 1.5 × IQR from the upper hinge, while the lower whisker extends to the minimum value no less than 1.5 × IQR from the lower hinge. Kaplan-Meier plot was generated by ggsurvplot implemented in R package survminer, and *P* values for comparing between risk groups were generated by log-rank test. For in vitro and in vivo experiments, unpaired *t* test and paired *t* test were used to analyze data between 2 groups. In vitro experiments were repeated to obtain standard deviations, and representative experiments are shown. Statistical significance was considered with a 2-tailed *P* value of less than 0.05. GraphPad Prism version 9.5.1 was used for analyses. Some images were created with Biorender (license number D3D32E84-0003).

### Data availability.

RNA-seq data of whole-blood transcriptomes from the recipients in the parent GoCAR study are available in the NCBI GEO database (GSE112927, before transplant; GSE120398, 3 months after transplant). RNA-seq and genotype data from the DICE project are available in the dbGaP under accession number phs001703. SNP array data generated from the GoCAR and CTOT-01/17 studies are available from the corresponding author upon request. Values for all data points found in graphs can be found in the Supporting Data Values file.

### Study approval.

For the GoCAR study, written informed consent was obtained from all participants from the individual clinical sites at the time of enrollment in the original study. IRB approval was obtained from all participating institutions (see list below for GoCAR and CTOT). For the CTOT study, written informed was obtained from all participants from the individual clinical sites at the time of enrollment in the original study. IRB approval was obtained from all participating institutions. Consent included the use of deidentified genetic data for research purposes and retrospective data reporting.

Participating institutions for GoCAR: Icahn school of Medicine at Mount Sinai, New York, New York, USA; University of Sydney, Westmead Hospital, Sydney, New South Wales, Australia; University of Wisconsin-Madison, Madison, Wisconsin, USA; Northwestern University, Northwestern Memorial Hospital, Chicago, Illinois, USA; University of Michigan, Ann Arbor, Michigan, USA; Massachusetts General Hospital–Brigham and Women’s Hospital, Harvard Medical School, Boston, Massachusetts, USA.

Participating institutions for CTOT: Icahn school of Medicine at Mount Sinai; University Hospitals of Cleveland, Cleveland Clinic Foundation, Cleveland, Ohio, USA; Cincinnati Children’s Hospital Medical Center, Cincinnati, Ohio, USA; Yale University School of Medicine, New Haven, Connecticut, USA; University of Manitoba, Children’s Hospital of Winnipeg, Winnipeg, Manitoba, Canada; Emory University Hospital, Emory Children’s Center, Atlanta, Georgia, USA.

## Author contributions

MCM and ZZ conceptualized the study. MCM obtained funding. PSH led the parent CTOT consortium and provided data and samples. PJO, LG, and AD were investigators in the GoCAR clinical study. RBC and IWG reported all study biopsies. ZS and ZZ designed the bioinformatic pipeline. ZY and WZ contributed data and bioinformatic analysis. KB led and performed the validation in vitro experimental work. AR and JP performed DNA extraction and sample preparation with KB and contributed to in vitro experiments. JCH, JSP, TDM, and BDK provided reagents, critical feedback on experiments and supervised analyses. MCM, ZZ, ZS, KB, and PSH drafted the manuscript. All coauthors contributed to editing and redrafting the submitted version of this manuscript.

## Supplementary Material

Supplemental data

Supplemental tables 4-15

Supporting data values

## Figures and Tables

**Table 2:**
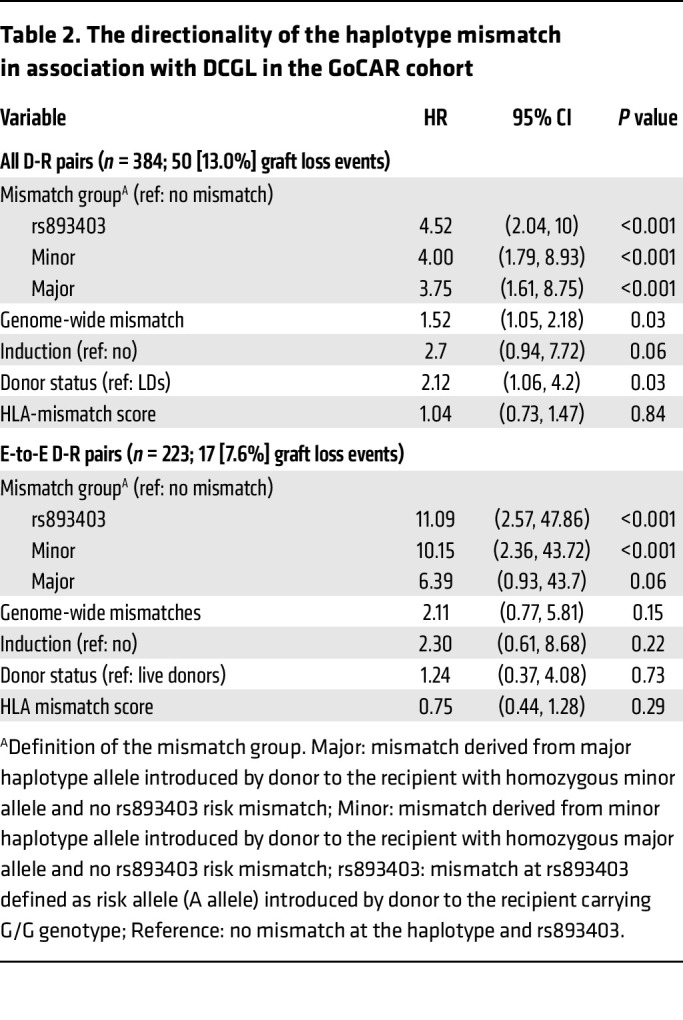
The directionality of the haplotype mismatch in association with DCGL in the GoCAR cohort

**Table 1:**
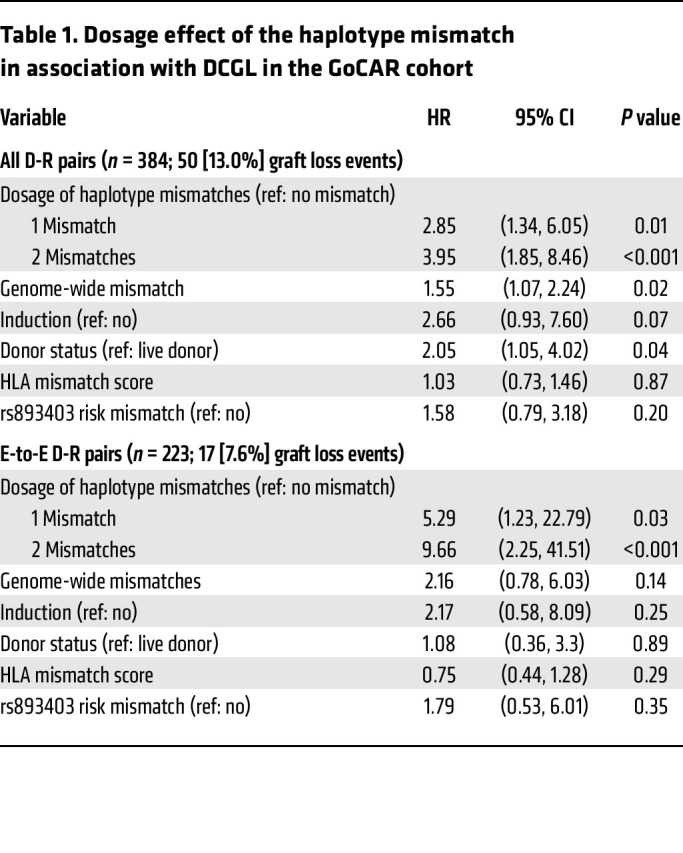
Dosage effect of the haplotype mismatch in association with DCGL in the GoCAR cohort

## Version 1 09/07/2023

In-Press Preview

## Version 2 11/01/2023

Electronic publication
