# Supplementary material for: Multiscale genetic architecture of donor-recipient differences reveals intronic LIMS1 mismatches associated with kidney transplant survival
Source: J Clin Invest. 2023 Nov 1;133(21):e170420. doi: 10.1172/JCI170420 (PMC10617779; doi:10.1172/JCI170420)
Supplement: Supplemental data [file jci-133-170420-s166.pdf]

Supplementary materials:
Multiscale genetic architecture of donor-recipient differences reveals intronic *LIMS1* locus mismatches associated with long-term renal transplant survival

Supplementary Figures

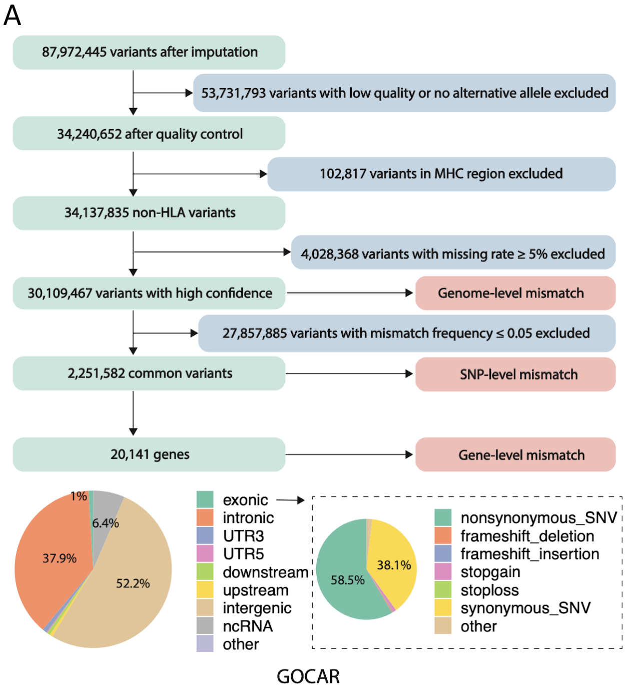

Figure S1. Quality control of the imputed genome-wide genotype data for the GoCAR (left) and CTOT (right) cohorts.

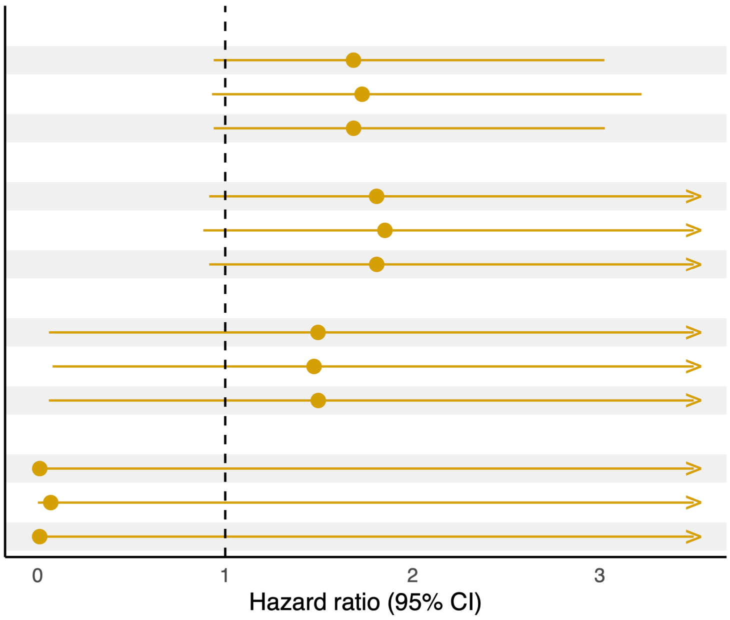

	P value	Hazard ratio (95% CI)
GOCAR univariate		
All SNPs	0.081	1.68 (0.94, 3.02)
Exonic SNPs	0.084	1.73 (0.93, 3.22)
Non−exonic SNPs	0.081	1.68 (0.94, 3.03)
GOCAR adjusted		
All SNPs	0.089	1.81 (0.91, 3.57)
Exonic SNPs	0.103	1.85 (0.88, 3.89)
Non−exonic SNPs	0.089	1.81 (0.91, 3.58)
CTOT univariate		
All SNPs	0.807	1.49 (0.06, 37.79)
Exonic SNPs	0.796	1.47 (0.08, 27.81)
Non−exonic SNPs	0.807	1.5 (0.06, 38.23)
CTOT adjusted		
All SNPs	0.48	0.01 (0, 3531.59)
Exonic SNPs	0.468	0.07 (0, 94.66)
Non−exonic SNPs	0.48	0.01 (0, 3798.95)

Figure S2. Association of different genome-wide D-R mismatch scores with DCGL in European-to-European transplants in GoCAR and CTOT. Genome-wide mismatch scores were calculated for all SNPs, exonic SNPs (SNPs located in exonic region), non-exonic SNPs (all SNPs minus exonic SNPs). Univariate and multivariable Cox regression (adjusting HLA mismatch score, induction therapy, and donor status) analyses were performed for both GoCAR and CTOT cohorts.

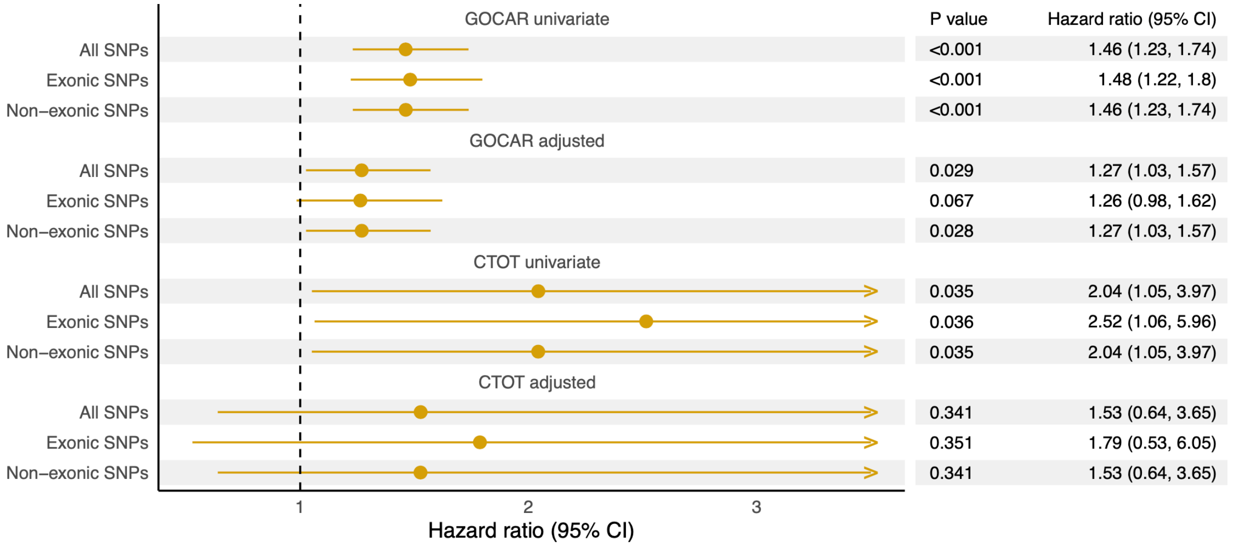

Figure S3. Association of different genome-wide D-R mismatch scores with DCGL in all transplants. Genome-wide mismatch scores were calculated for all SNPs, exonic SNPs (SNPs located in exonic region), non-exonic SNPs (all SNPs excluding exonic SNPs). Univariate and multivariable Cox regression (adjusting HLA mismatch score, induction therapy, and donor status) analyses were performed for both GoCAR and CTOT cohorts. HLA 4-antigen (A, B, DR, and DQ) mismatch score was used in GoCAR and 6-antigen (A, B, C, DP, DR, and DQ) mismatch score in CTOT.

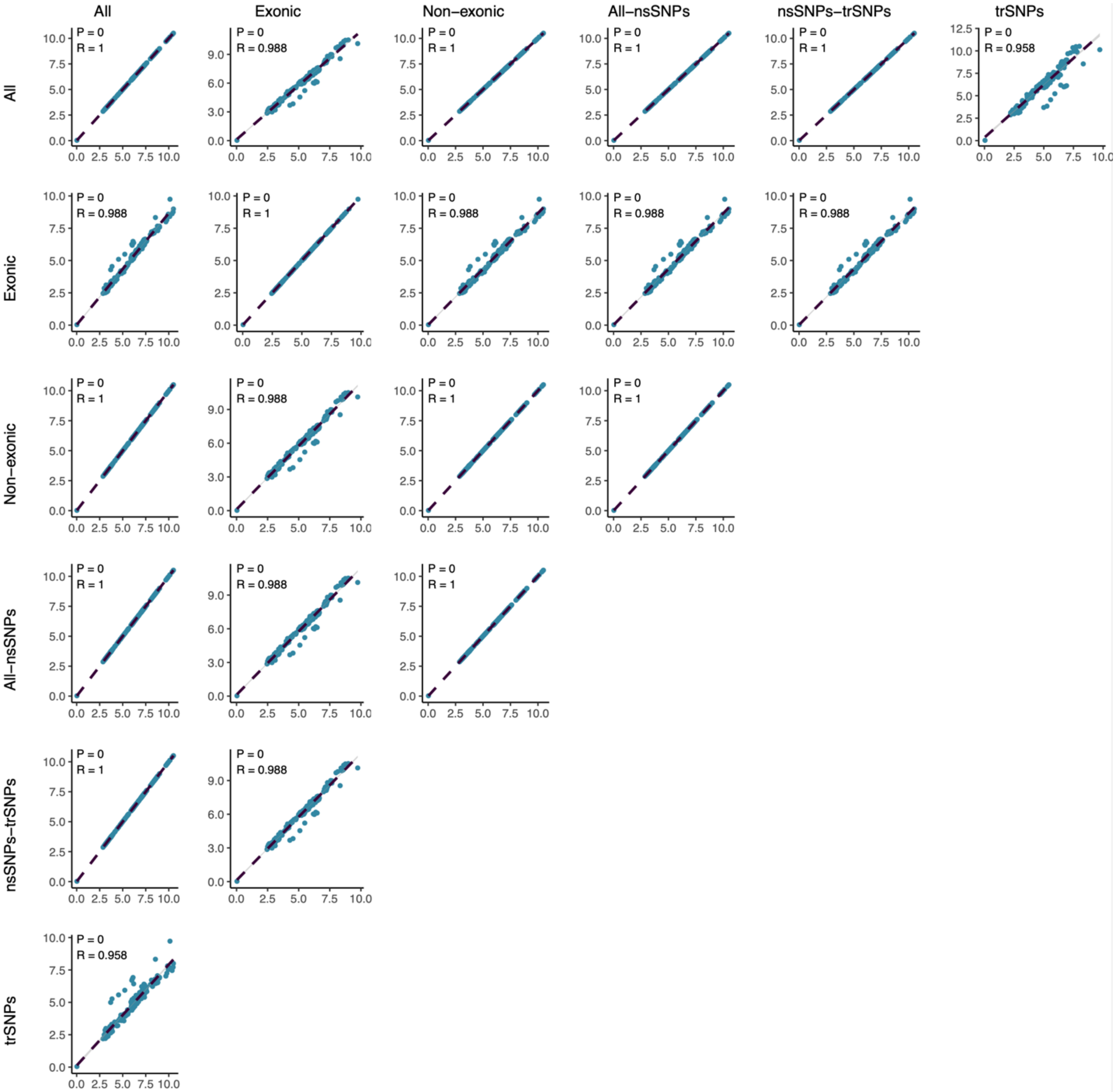

Figure S4. Correlation among D-R mismatch scores defined within different genomic scopes in GoCAR. SNP-level any mismatches were summed for all SNPs, exonic SNPs, non-exonic SNPs (all SNPs excluding exonic SNPs), all-nsSNPs (all SNPs excluding non-synonymous SNPs), nsSNPs-trSNPs (non-synonymous SNPs excluding transmembrane SNPs), and trSNPs (transmembrane SNPs) as raw counts, and normalized by their scope-specific IQR, respectively.

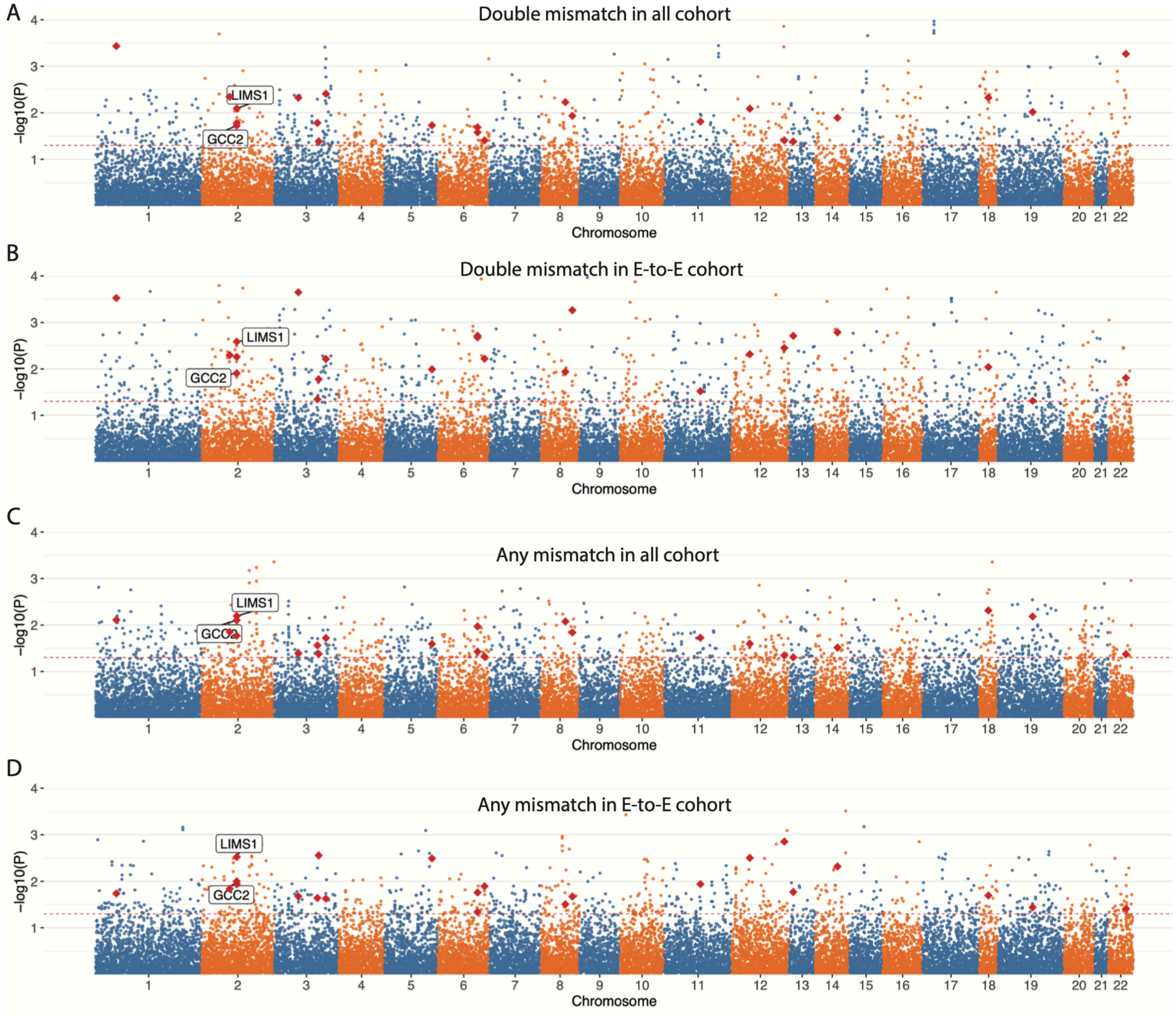

Figure S5. Manhattan plots for genome-wide association tests of gene-level mismatch scores with DCGL in GoCAR using different models: (**A**) double mismatch in all cohort, (**B**) double mismatch in the sub-cohort of E-to-E D-R pairs, (**C**) any mismatch in all cohort, and (**D**) any mismatch in the sub-cohort of E-to-E D-R pairs. The 23 genes consistently showing significant association signals (nominal P ≤ 0.05) are highlighted in red diamond.

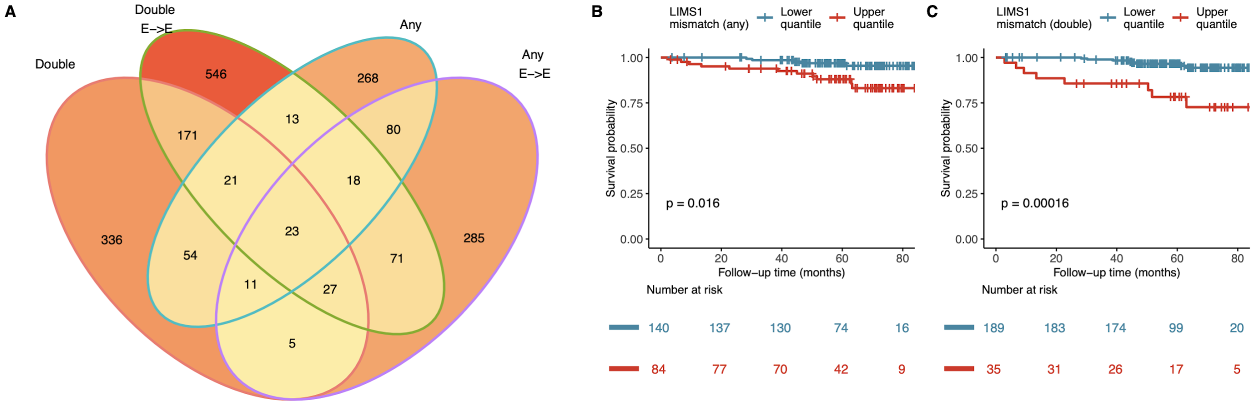

Figure S6. Gene-level mismatches associated with graft loss. (**A**) Venn diagram shows the number of genes identified with mismatch score significantly associated with DCGL (nominal p <= 0.05) from four different analyses: double mismatch or any mismatch (definition in Figure 1 and Methods) for the whole GoCAR cohort or the subset of European-to-European (E-to-E) D-R pairs. In GoCAR E-to-E D-R pairs, Kaplan-Meier plots show the graft survival curves for equally dichotomized groups of mismatch scores at *LIMS1* locus, where mismatch scores were defined as "any mismatch" in (**B**) and "double mismatch" in (**C**). P-values were derived from log-rank tests in comparison of upper quantile versus lower quantile.

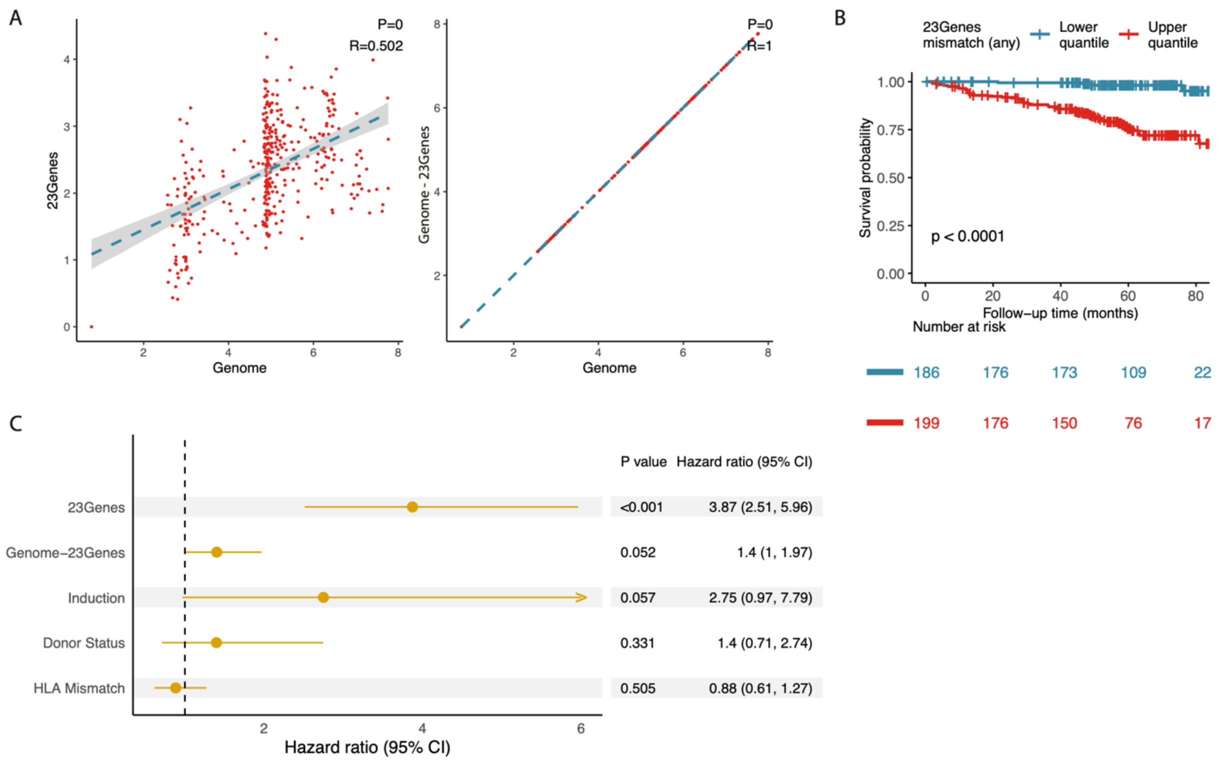

Figure S7. Summary of mismatch score of the 23 candidate genes was significantly related to DCGL. (A) The Correlation of the summary score of the 23 genes and other gene regions with genome-wide mismatch score. (B) Survival curve of the patients stratified by the 23 genes' mismatch score grouped by mean value. (C) Forest plot of the hazard ratio of 23 genes' mismatch score to DCGL adjusted by mismatch score of the other genome regions, Induction therapy, donor status and HLA mismatch.

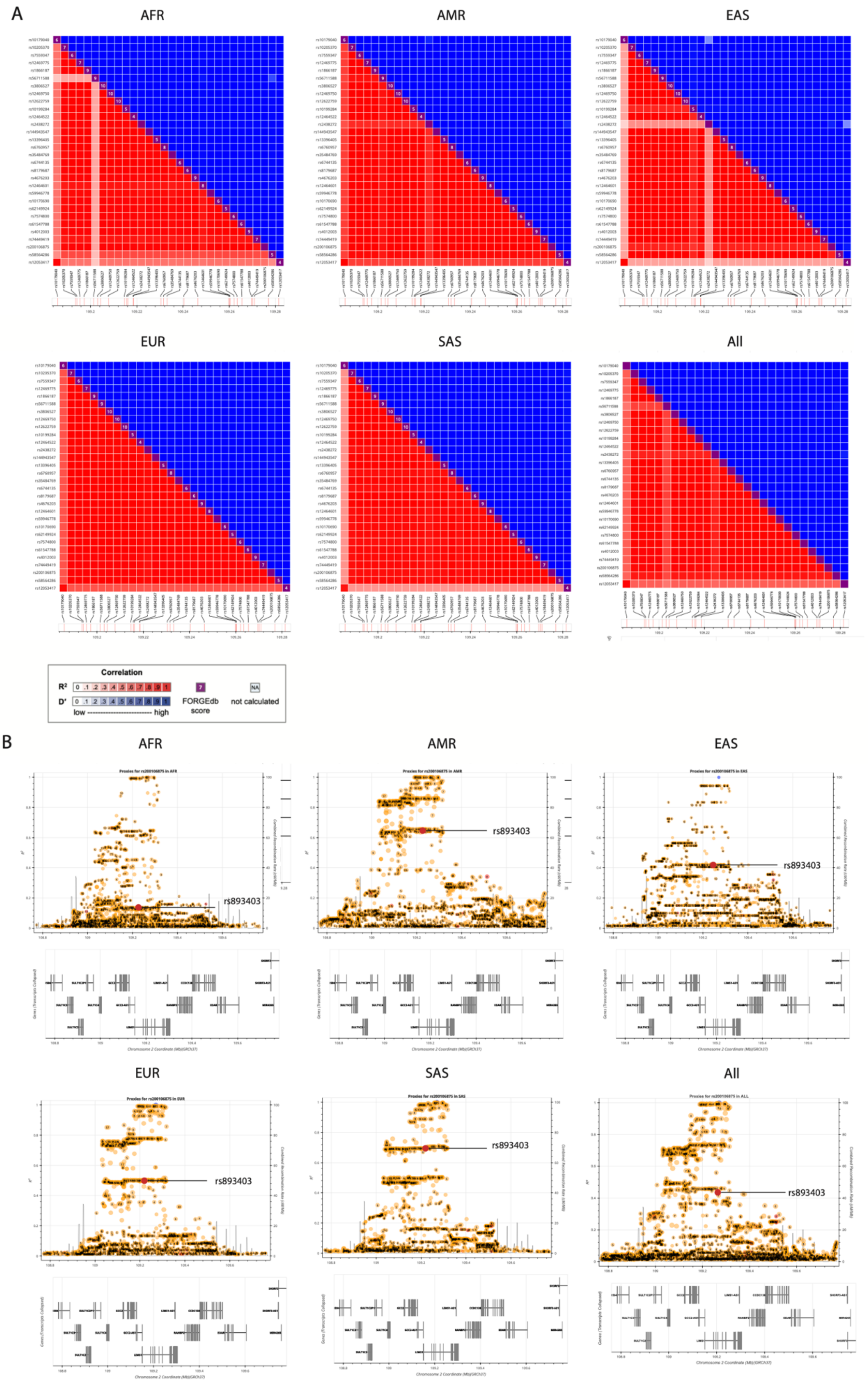
A
AFR
AMR
EAS
EUR
SAS
All
Correlation
R²
D'
low
high
FORGEdb score
NA
not calculated
B
AFR
AMR
EAS
EUR
SAS
All
rs893403
rs893403
rs893403
rs893403
rs893403
rs893403

Figure S8. Linkage disequilibrium between 30 top candidate SNPs and rs893403 within the LIMS1 locus. (**A**) The LD metrics R^2 (red) and D' (blue) were calculated based on the genotype data from all the continental populations of the 1000 Genomes Project (hg19) using LDlink[1]. (**B)** The LD structure of the region surrounding the 30 candidate SNPs and rs893403 generated by LDlink, displayed as the R^2 value with a representative SNP rs200106875 of the haplotype of 30 candidate SNPs. The SNP rs893403 is highlighted by a red dot, clearly located in a distinct haplotype other than the 30-SNP haplotype. Gene models are shown below.

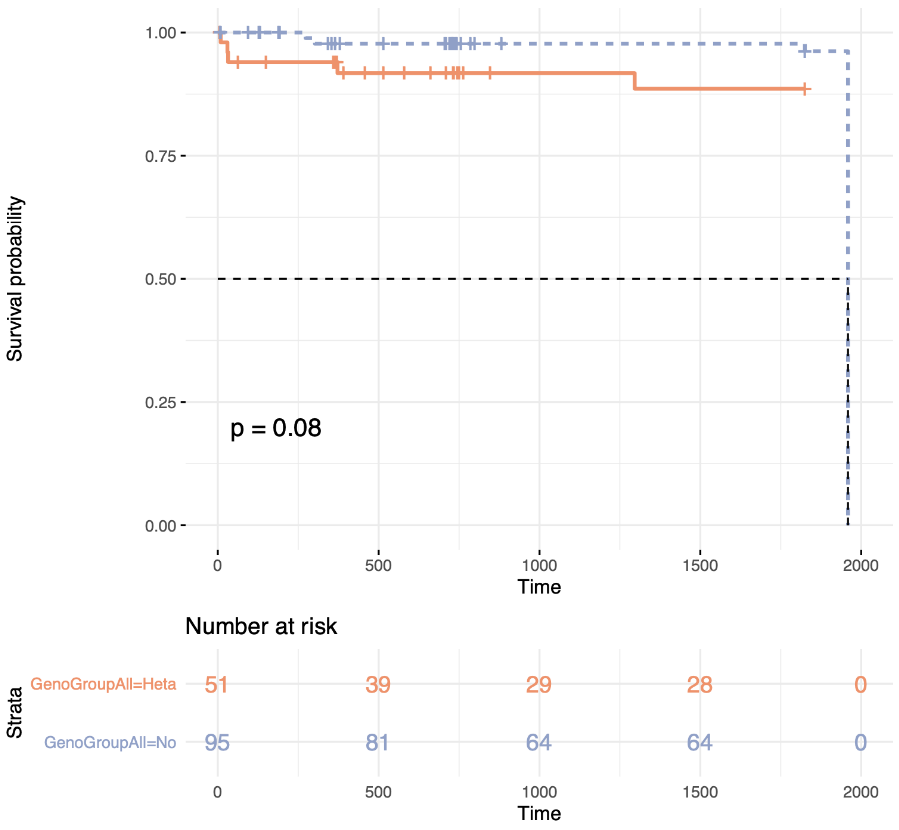

Figure S9. Kaplan-Meier curves of DCGL for the CTOT patients grouped by presence and absence of any D-R mismatch of the identified LIMS1 haplotype. P-value was derived from log-rank test.

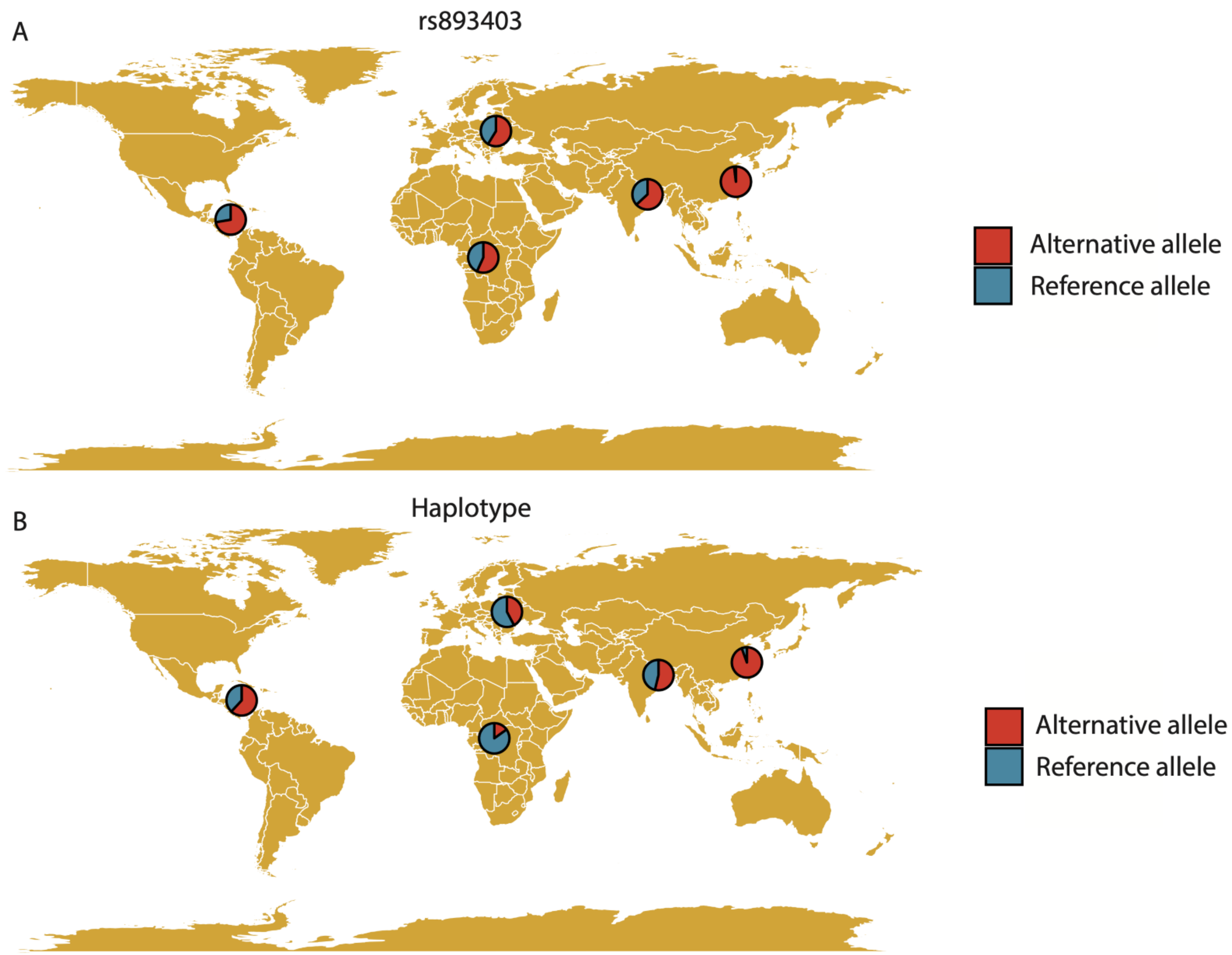

Figure S10. Allele frequency of rs893403 (A) and haplotype (B) in different major ancestral populations. The allele frequencies were retrieved from 1000 Genomes project deep whole-genome sequencing data. The MAF of the haplotype was calculated as the mean value of the MAF of the 30 SNPs.

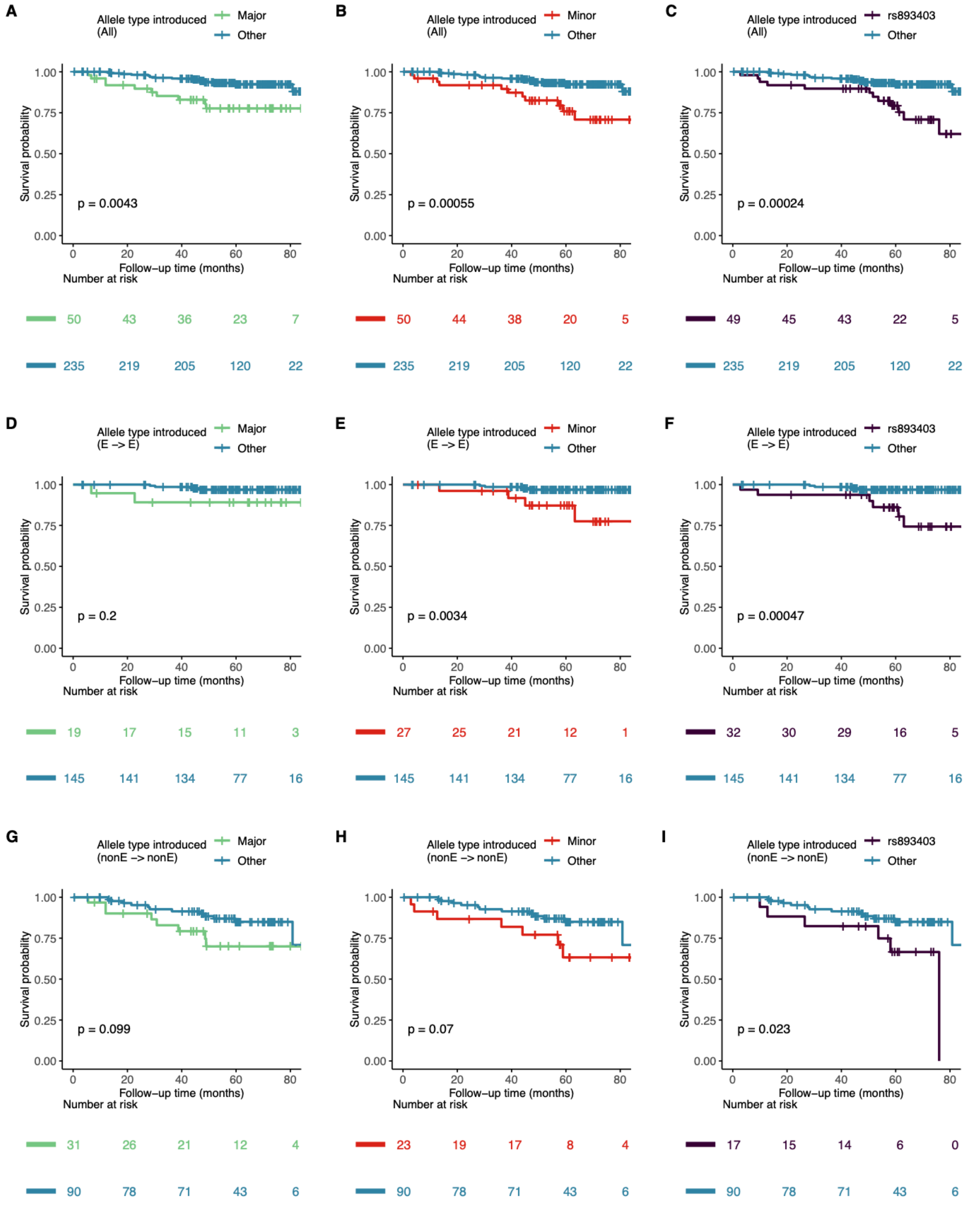

Figure S11. Directionality of the haplotype mismatch associated with DCGL in GoCAR. Kaplan-Meier plots show the graft survival curves grouped by the directionality of the mismatches at the haplotype and the rs893403 risk mismatch in all (**A-C**), E-to-E (**D-F**), and non-E-to-E (**G-I**) D-R pairs. Major: mismatch derived from major haplotype allele introduced by donor to the recipient with homozygous minor allele

and no rs893403 risk mismatch; Minor: mismatch derived from minor haplotype allele introduced by donor to the recipient with homozygous major allele and no rs893403 risk mismatch; rs893403: mismatch at rs893403 defined as risk allele (A allele) introduced by donor to the recipient carrying G/G genotype; Other: no mismatch at the haplotype and rs893403. P-values were derived from log-rank tests.

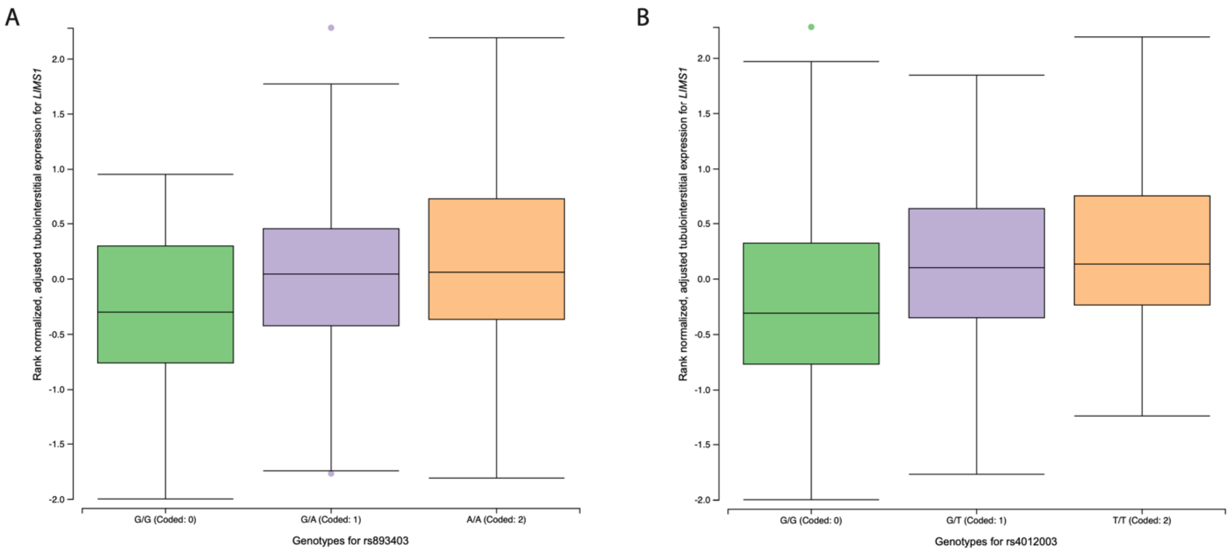

Figure S12. eQTL data of rs893403 (A) and the haplotype (represented as one of the candidate SNP rs4012003) (B) in tubulointerstitial tissue from NephQTL[2].

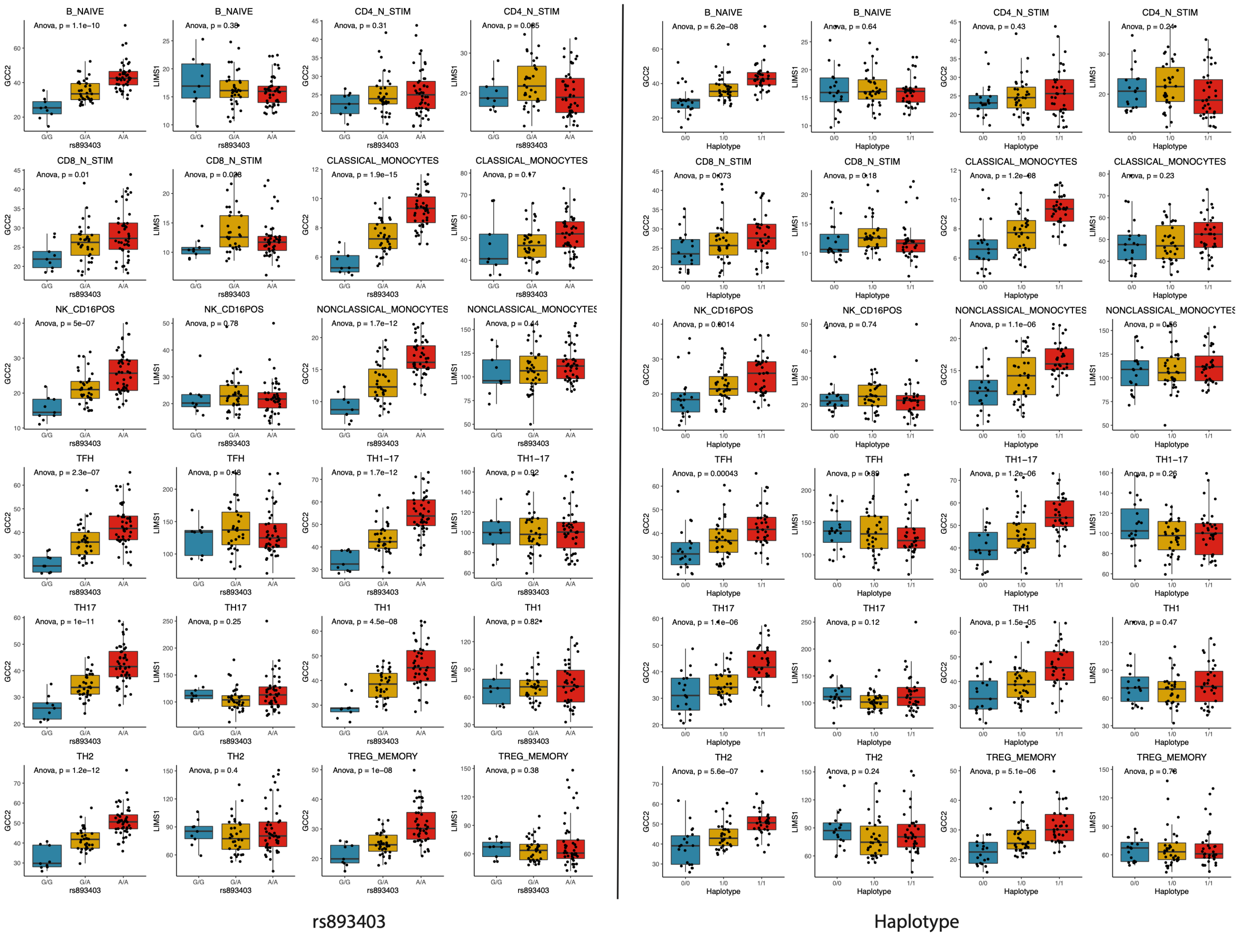

Figure S13. eQTL analysis of GCC2 and LIMS1 using DICE data. Box plots show the distribution of *GCC2* and *LIMS1* expression within each genotype group of rs893403 (left panel) and the haplotype (right panel) in 12 out of the 15 immune cell types from the DICE cohort (naive Treg, naive CD4⁺ T cell and naive CD8⁺ are shown in Figure 6A. The significance of the association between expression level and the genotype is indicated by the p-value derived from ANOVA.

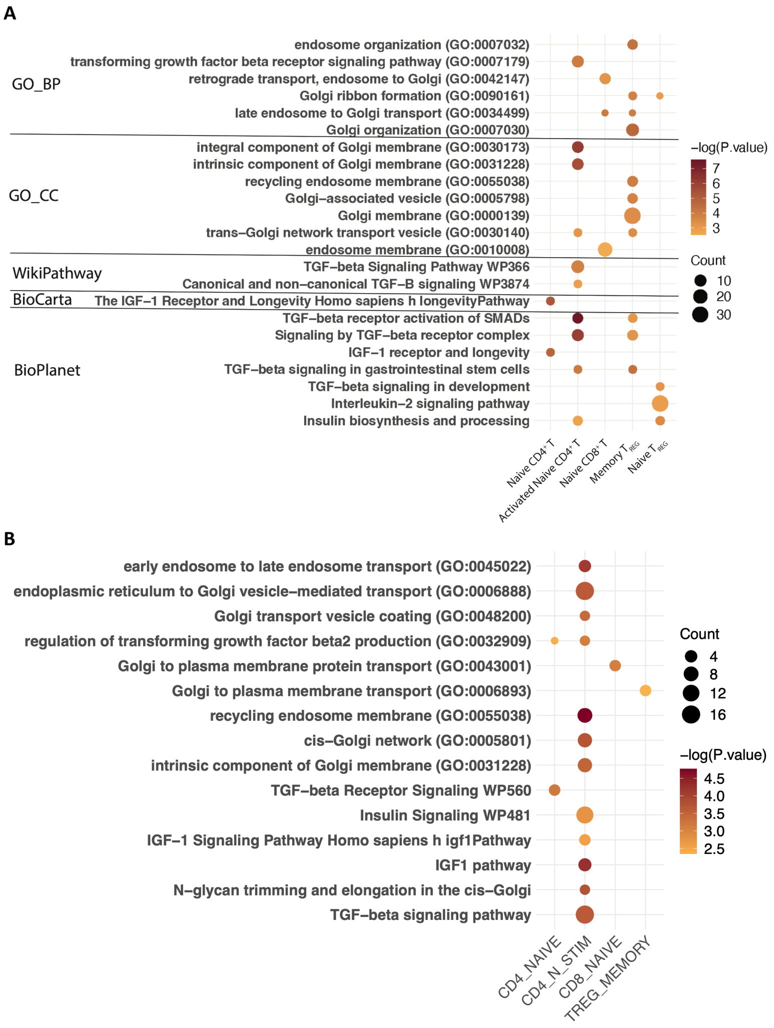

Figure S14. Enriched functions of DEGs in relevant immune cell types from the DICE cohort. Differentially expressed genes (DEGs) were identified by associating with the number of risk alleles of rs893403 (**A**) and the haplotype (**B**) with a nominal $p \leq 0.05$.

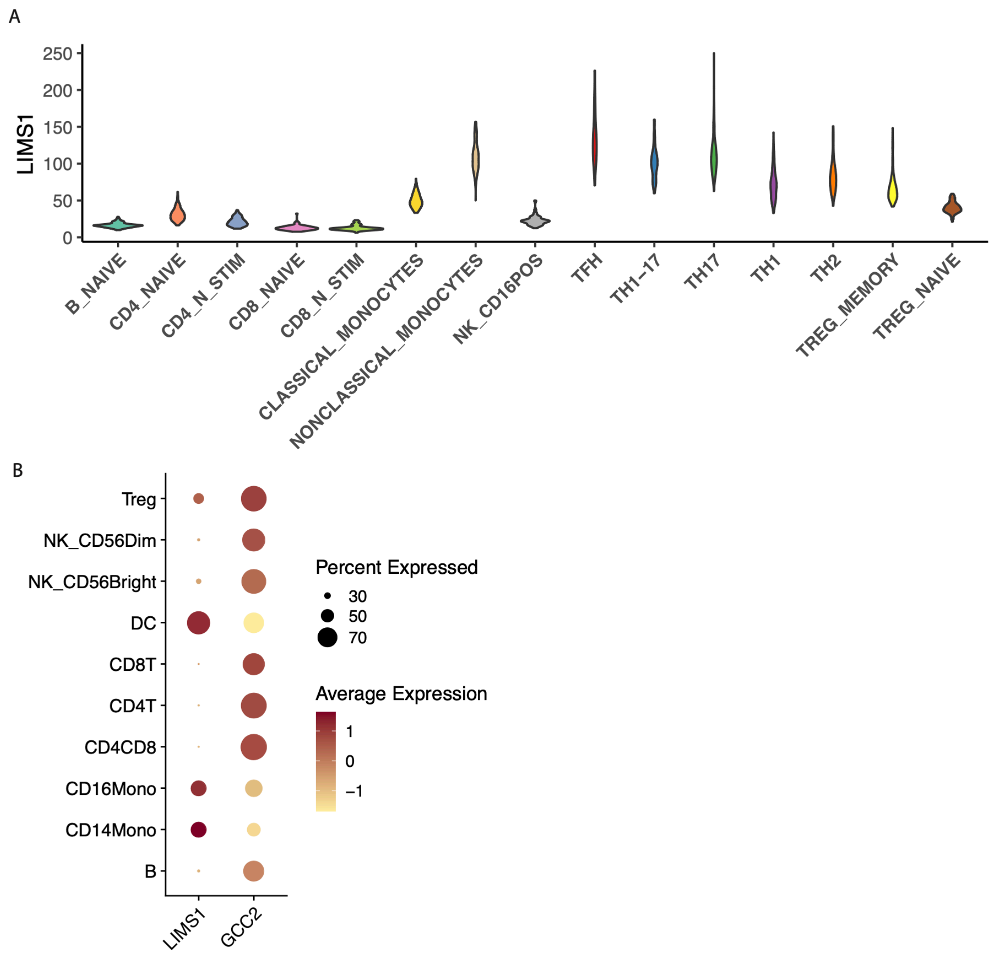

Figure S15. Expression levels of *LIMS1* and *GCC2* in different immune cell types from published bulk and single-cell RNA-seq datasets. (**A**) Distribution of LIMS1 expression in sorted PBMC subtypes from the DICE cohort. (**B**) Expression levels of *LIMS1* and *GCC2* in the PBMC single cell data.

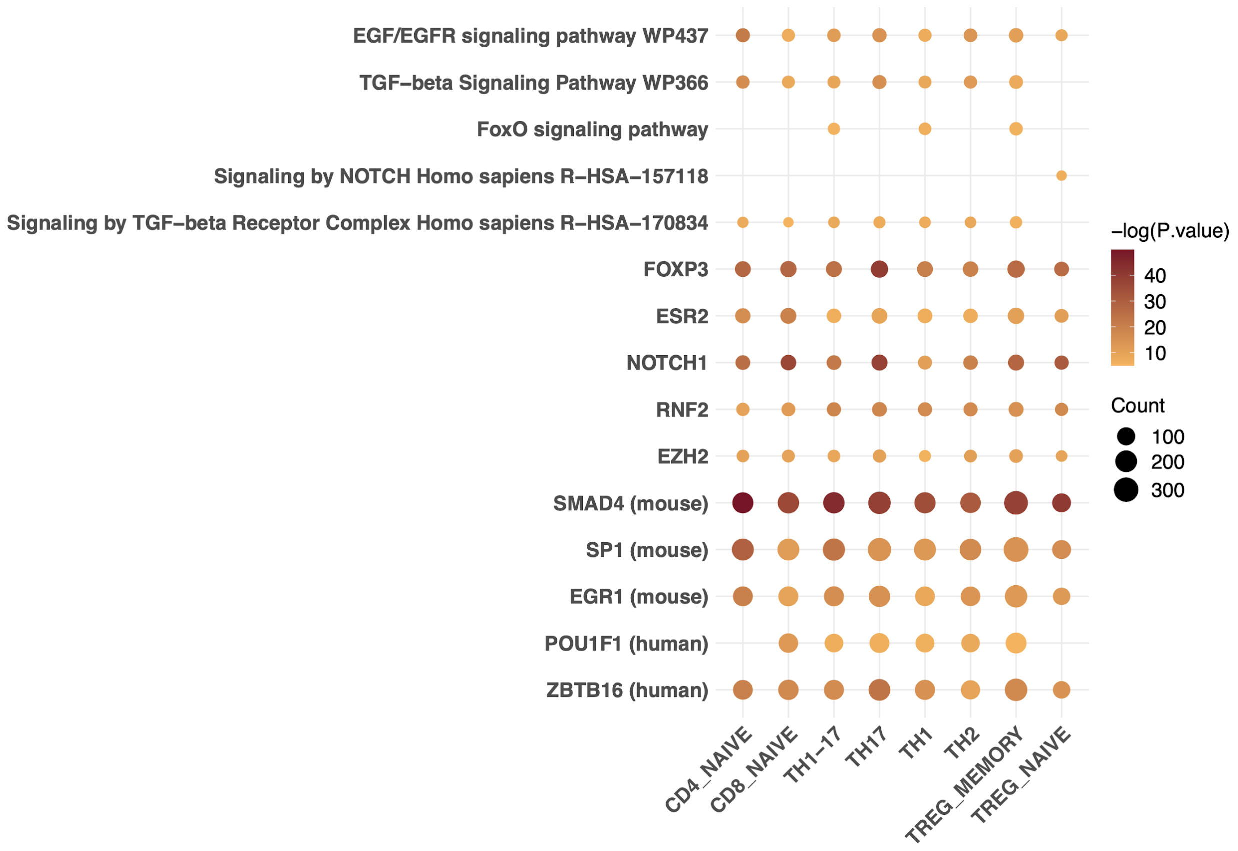

Figure S16. Enriched functions and transcription factors of *GCC2* co-expressed genes in multiple T cell subtypes from the DICE cohort.

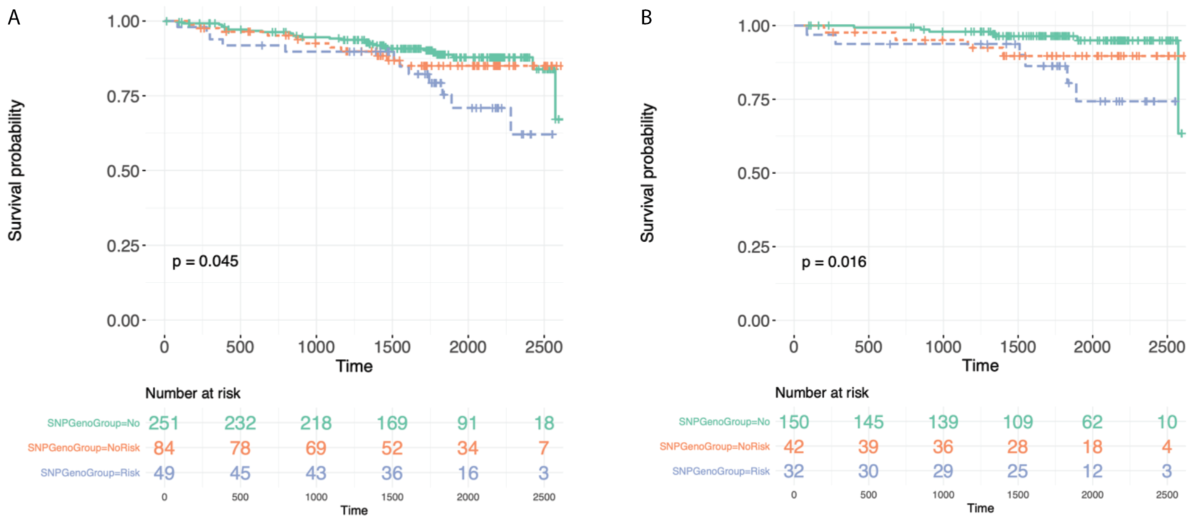

Figure S17. Survival curves of DCGL in all patients(A) and E-to-E (B) of the GoCAR cohort grouped by rs2460944 risk allele (A allele) introduced by donor.

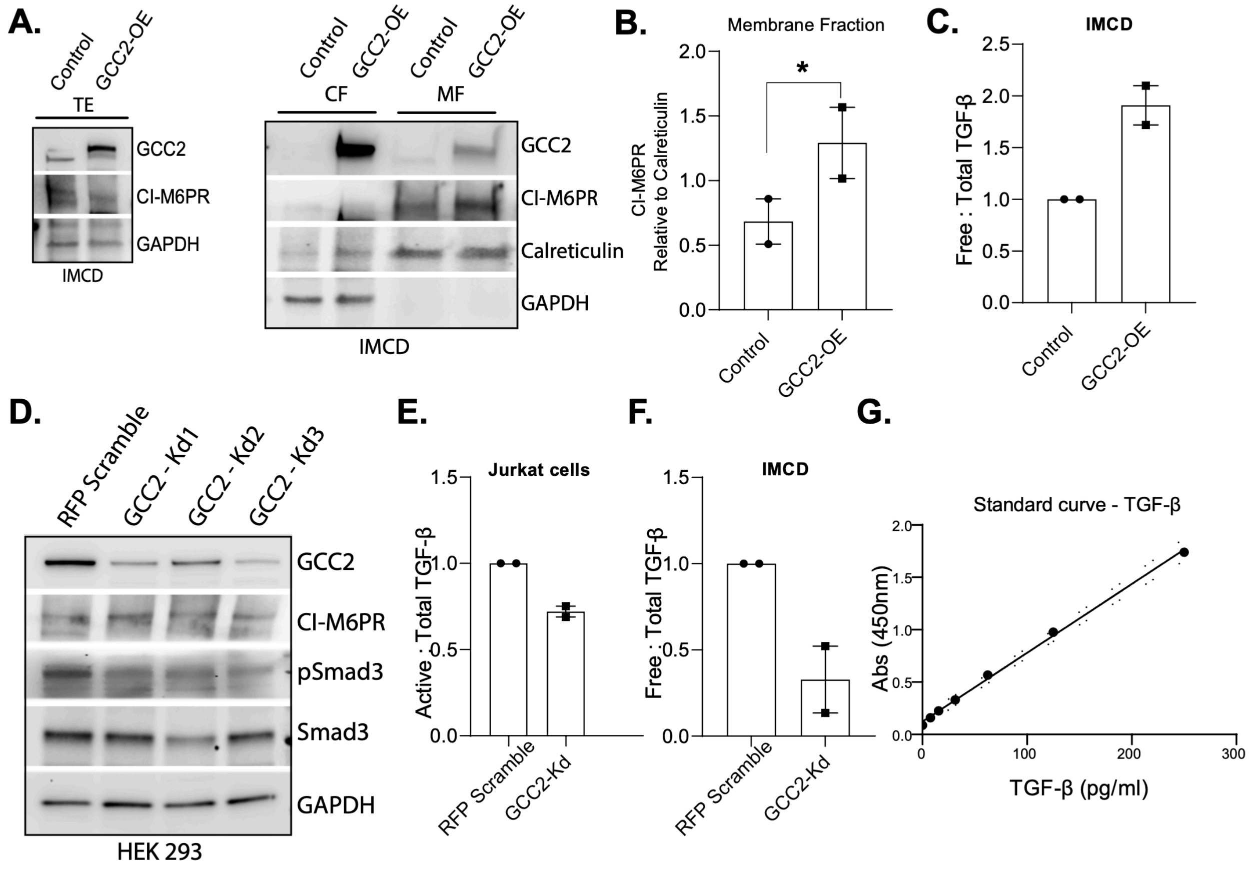

Figure S18. *GCC2* modulate generation of active TGFB1 and downstream signaling in lymphocytes and epithelial cell lines. (A-C) We overexpressed either a GFP-GCC2 (GCC2-OE) or a GFP Control construct and confirmed overexpression in IMCD cells, followed by extraction of protein lysates, subcellular fractionation, and immunoblotting (n=2 sets). (**A**) Representative immunoblots of cellular fractions probed for GCC2, CI-M6PR, Calreticulin, and GAPDH are displayed for GCC2-OE- vs GFP-control-IMCD cells. (**B**) Dot plots show corresponding densitometric quantifications of CI-M6PR in the MFs (normalized to Calreticulin) from two experiments. (**C**) Dot plot shows corresponding ratios of active (LAP cleaved) to total TGFB1 levels (both in pg/ml normalized to Control in each experiment and analyzed by paired t-test) in GCC2-OE and Control IMCD supernatants assayed by ELISA after 24 hours serum starvation. **(D-G)** To further investigate the cellular role of GCC2, we used Lentiviral-ShRNA mediated knockdown of GCC2 (3 sequences named GCC-Kd1, -Kd2, -Kd3 respectively), and compared this with a scramble shRNA (RFP Scramble) infected control lentivirus in Jurkat T-cells, HEK-293Tcells, or IMCD cell lines. **(D)** Representative Immunoblots probed for GCC2 (normalized to GAPDH) confirmed GCC2 knockdown in HEK 293 cells, and total lysates were immunoblotted for Phosphorylated and total SMAD3. Dot plots show ratios of active (LAP cleaved) to total TGFB1 levels (both in pg/ml normalized to scramble in each experiment and analyzed by paired T-test) in GCC2-knockdown vs. Scramble-infected supernatants from Jurkat T-cell (**E**) and IMCD cells (**F**) assayed by ELISA after 24 hours serum starvation (n=2 sets of experiments each). (**G**) A representative standard curve is generated during TGFB1-ELISA experiments [Ratios paired t-test unless specified; *: P<0.05; CI-M6PR: Cation independent mannose-6-Phosphate receptor; MF and CF: membrane and cytoplasmic fraction of lysate; IMCD: rat inner medullary collecting duct cells; TGF-β: TGFB1].

Supplementary tables

Table S1. Demographic and clinicopathologic characteristics of donors and recipients with genome-wide genotype data in the GoCAR and CTOT1/17 cohorts.

Variable	GOCAR D-R pairs with genotype (n = 385) [a]	CTOT D-R pairs with genotype (n = 146) [b]	P-value[c]
<u>**Recipient**</u>			
**Death censored graft loss** (years)			
mean ± SD; median (range)	4.6 ± 1.7; 4.9 (0.04, 7.3)	3.7 ± 1.8; 5.0 (0.0, 5.4)	**<0.001**
# events (%)	50 (13.0%)	9 (6.2%)	**0.03**
**Age** (years), mean ± SD; median (range)	49.9 ± 13.5; 50 (18, 83)	43.5 ± 18.2; 47.5 (2, 89)	**<0.001**
**Gender**, male, n (%)	257 (66.8%)	88 (60.3%)	0.19
**Genetic ancestry**[d], n (%)			0.40
African American	70 (18.2%)	33 (22.6%)	
Asian	13 (3.4%)	2 (1.4%)	
Caucasian	235 (61.0%)	90 (61.6%)	
Hispanic	67 (17.4%)	21 (14.4%)	
**HLA mismatch score**[e], n (%)	2.0 ± 1.0	2.8 ± 1.0	**0.01**[f]
**Induction**, n (%)			0.14
No induction	78 (20.3%)	38 (26.0%)	
Non-depletional (IL2 antagonist)	130 (33.8%)	54 (37.0%)	
Depletional (Thymoglobulin or Campath)	177 (46.0%)	54 (37.0%)	
<u>**Donor**</u>			
**Age** (years), mean ± SD; median (range)	42.6 ± 14.7; 45 (3, 73)	39.3 ± 12.2; 38 (6, 65)	**0.01**
**Gender**, male, n (%)	196 (50.9%)	63 (43.2%)	0.14
**Genetic race**, n (%)			**0.006**
African American	33 (8.6%)	28 (19.2%)	
Asian	7 (1.8%)	2 (1.4%)	
Caucasian	293 (76.1%)	94 (64.4%)	
Hispanic	52 (13.5%)	22 (15.1%)	

**Donor type,** live donor, n (%)	194 (50.4%)	123 (84.8%)	**<0.001**

[a]: Genome-wide genotype data is available for 385 donor-recipient (D-R) pairs from the parent GOCAR study after data processing and quality control detailed elsewhere[3].

[b]: Genome-wide genotype data is available for 146 donor-recipient (D-R) pairs from the parent CTOT study after data processing and quality control detailed elsewhere [ref KI].

[c]: P-value was calculated from unpaired t-test for continuous variables and from Fisher's exact test for categorical variables unless otherwise specified. Bold p-value < 0.05.

[d]: Genetic ancestry was inferred from genome-wide genotype data and considered more accurate than self-reported race[3].

[e]: HLA mismatch score was derived from 2-digit HLA allele typing. Following previous reports for GOCAR[3-5], the raw mismatch score (scaling from 0 to 6) was categorized into: 0 (no mismatches), 1 (1-2 mismatches), 2 (3-4 mismatches), and 3 (5-6 mismatches); while for the CTOT cohort, the raw mismatch score (scaling from 0 to 6) was used. In subsequent statistical analyses, this variable was used as numeric covariate in regression models.

[f]: In order to calculate the p-value, the raw HLA mismatch score used in CTOT was hereby categorized in the same way as GOCAR so that the HLA mismatch scores originally defined on different scales in the two cohorts are comparable. The p-value was calculated by Fisher's exact test.

Table S2. Causes of DCGL in the GoCAR cohort.

Cause of graft loss (n = 50)	CAI/IFTA/Chronic Rejection	Acute Rejection	Infections*	Others	PNF
**DCGL**, n (%)	18 (36%)	8 (16%)	11 (22%)	11 (22%)	2 (4%)

*Includes BK virus disease

Table S3. Statistics of genome level mismatches between donor-recipient pairs.

	GoCAR	CTOT
Whole genome	1,280,474.86 ± 335,138.36	233,365.3 ± 97,270.23
Non-exonic SNPs	1,272,112.08 ± 332,958.53	230,786.45 ± 96,425.89
Exonic SNPs	8,362.78 ± 2,205.68	2,578.85 ± 873.09
Non-synonymous SNPs	4,058.77 ± 1078.77	1,449.05 ± 468.12

Table S4. Top candidate genes with gene-level D-R mismatches associated with DCGL in GoCAR.

Table S5. Association of *LIMS1* mismatch with DCGL using univariate and multivariable Cox regression analysis in CTOT.

Variable	HR	95% CI	P value
**Univariate analysis: D-R pairs of all ancestries** (n = 146; 9 [6.2%] graft loss events)			
LIMS1 gene level mismatch (any mismatch) (ref: no mismatch)	4.08	(1.21, 13.79)	**0.02**
**Multivariable analysis: D-R pairs of all ancestries** (n = 146; 9 [6.2%] graft loss events)			
LIMS1 gene level mismatch (any mismatch) (ref: no mismatch)	6.08	(1.46, 25.36)	**0.01**
Genome-wide mismatch	1.73	(0.66, 4.55)	0.26
Donor status (ref: Living donor)	0.33	(0.07, 1.50)	0.15
HLA mismatch score	1.20	(0.78, 1.83)	0.41

Table S6. Association of *LIMS1* gene-level mismatch with DCGL in GoCAR and CTOT using multivariable Cox regression.

Variable[a]	HR	95% CI	P value
**GoCAR**			
***LIMS1* gene-level mismatch**	2.16	(1.29, 3.62)	**0.003**
**Genome-wide mismatch**	1.25	(1.00, 1.55)	**0.049**
**HLA mismatch score (4 antigens)**	0.98	(0.84, 1.15)	0.836
**CTOT**			
***LIMS1* gene-level mismatch**	5.35	(1.26, 22.71)	**0.023**
**Genome-wide mismatch**	1.89	(0.75, 4.76)	0.180
**HLA mismatch (6 antigens)**	1.01	(0.79, 1.29)	0.938

[a]: Multivariable Cox models were adjusted with HLA 4-antigen (A, B, DR, and DQ) mismatch score for GoCAR and HLA 6-antigen (A, B, C, DP, DQ, and DR) mismatch score for CTOT along with other covariates. Mismatch related variables were shown.

Table S7. Association of *LIMS1* gene-level mismatch, rs893403, and haplotype mismatch with DCGL in 385 GoCAR patients using muti-variable Cox regression analysis adjusted by the presence of anti-HLA DSA.

Variable[a]	HR	95% CI	P value
LIMS1 gene-level mismatch	2.21	(1.32, 3.70)	**0.002**
Genome-wide mismatch	1.27	(1.01,1.60)	**0.042**
Donor specific antibody	3.31	(1.56, 7.02)	**0.002**
rs893403 risk mismatch	2.23	(1.15, 4.30)	**0.017**
Genome-wide mismatch	1.30	(1.04, 1.64)	**0.023**
Donor specific antibody	3.05	(1.45, 6.42)	**0.003**
Haplotype mismatch	3.00	(1.68, 5.37)	**<0.001**
Genome-wide mismatch	1.34	(1.06, 1.70)	**0.016**
Donor specific antibody	3.51	(1.65, 7.47)	**0.001**

[a]: Cox model adjusted by genome-wide mismatch, donor specific antibody, induction therapy, donor status and HLA mismatch.

Table S8. Top candidate variants at the *LIMS1* locus with D-R mismatches associated with graft loss.

Table S9. Association of identified *LIMS1* haplotype with DCGL in CTOT using multivariable Cox model.

Variable[a]	HR	95% CI	P-value
**D-R pairs of all ancestries** (n = 146; 9 [6.2%] graft loss events)			
Any haplotype mismatch (ref: no mismatch)	4.79	(1.03, 22.14)	**0.04**
Genome-wide mismatch	1.87	(0.93, 3.74)	0.08
Donor status (ref: Living donor)	0.75	(0.06, 1.30)	0.11
HLA mismatch score	1.04	(0.70, 1.55)	0.83

[a]: Induction therapy was excluded from adjusted covariates because the model would have not converged when including the variable.

Table S10. The directionality of the haplotype mismatch in association with DCGL in the non-E-to-E GoCAR cohort

Variable[a]		HR	95% CI	P value
**Non-E-to-E D-R pairs (n = 161; 33 [20.5%] graft loss events)**				
**Mismatch group[a] (ref: no mismatch)**				
	**rs893403**	2.83	(1.02, 7.85)	**0.04**
	**Minor**	1.92	(0.71, 5.2)	0.2
	**Major**	2.27	(0.84, 6.12)	0.1
**Genome-wide mismatch**		1.16	(0.72, 1.86)	0.54
**Induction (ref: No)**		3.56	(0.47, 26.95)	0.22
**Donor status (ref: LDs)**		2.56	(0.97, 6.77)	0.06
**HLA-mismatch score**		1.24	(0.76, 2.02)	0.39

[a]: Definition of the mismatch group. Major: mismatch derived from major haplotype allele introduced by donor to the recipient with homozygous minor allele and no rs893403 risk mismatch; Minor: mismatch derived from minor haplotype allele introduced by donor to the recipient with homozygous minor allele and no rs893403 risk mismatch; rs893403: mismatch at rs893403 defined as risk allele (A allele) introduced by donor to the recipient carrying G/G genotype; Reference: no mismatch at the haplotype and rs893403.

Table S11. Correlation of *LIMS1* and haplotype mismatch score with subclinical and clinical TCMR episodes

Variable[a]	OR	95% CI	P-value
All cohort			
LIMS1 mismatch score	1.16	(0.60, 2.22)	0.655
Haplotype mismatch score	1.11	(0.53, 2.23)	0.782
E-to-E cohort			
LIMS1 mismatch score	1.41	(0.55, 3.51	0.460
Haplotype mismatch score	1.70	(0.62, 4.54)	0.292

[a] Adjusted by genome-wide mismatch, induction therapy donor status and donor age.

Table S12. Correlation of number of haplotype minor alleles in the donor and number of haplotype mismatches with 12-month CADI or Ci+Ct score.

Variable[a]	OR	95% CI	p value
CADI 12 month			
All cohort			
Number of minor alleles in donor	1.43	(0.93, 2.23)	0.109
Number of mismatches between donor and recipient	1.78	(1.08, 2.97)	**0.025**
E-to-E cohort			
Number of minor alleles in donor	2.58	(1.36, 5.05)	**0.004**
Number of mismatches between donor and recipient	2.24	(1.10, 4.69)	**0.028**
Ci+Ct 12 month			
All cohort			
Number of minor alleles in donor	1.50	(0.97, 2.34)	0.070
Number of mismatches between donor and recipient	1.57	(0.96, 2.57)	0.072
E-to-E cohort			
Number of minor alleles in donor	3.00	(1.60, 5.89)	**0.001**
Number of mismatches between donor and recipient	1.67	(0.85, 3.28)	0.134

[a]Ordinal Logistic regression adjusted by genome-wide mismatch, induction therapy donor status and donor age.

Table S13. *GCC2* expression was associated with SNP rs893403 and the haplotype genotype in multiple blood cell types from healthy individuals in the DICE data[6].

Table S14. List of SNPs in high LD with SNP rs893403 and located at peak regions of the *LIMS1* locus in the kidney scATAC-seq data[7].

Table S15. List of SNPs in high LD with the haplotype and located at peak regions of the *LIMS1* locus the kidney scATAC-seq data[7].

References

1 Machiela, M. J. & Chanock, S. J. LDlink: a web-based application for exploring population-specific haplotype structure and linking correlated alleles of possible functional variants. *Bioinformatics* **31**, 3555-3557, doi:10.1093/bioinformatics/btv402 (2015).

2 Gillies, C. E. *et al.* An eQTL Landscape of Kidney Tissue in Human Nephrotic Syndrome. *Am J Hum Genet* **103**, 232-244, doi:10.1016/j.ajhg.2018.07.004 (2018).

3 Zhang, Z. *et al.* Genome-wide non-HLA donor-recipient genetic differences influence renal allograft survival via early allograft fibrosis. *Kidney Int* **98**, 758-768, doi:10.1016/j.kint.2020.04.039 (2020).

4 O'Connell, P. J. *et al.* Biopsy transcriptome expression profiling to identify kidney transplants at risk of chronic injury: a multicentre, prospective study. *Lancet* **388**, 983-993, doi:10.1016/S0140-6736(16)30826-1 (2016).

5 Zhang, W. *et al.* A Peripheral Blood Gene Expression Signature to Diagnose Subclinical Acute Rejection. *J Am Soc Nephrol* **30**, 1481-1494, doi:10.1681/ASN.2018111098 (2019).

6 Kent, W. J. *et al.* The human genome browser at UCSC. *Genome Res* **12**, 996-1006, doi:10.1101/gr.229102 (2002).

7 Sheng, X. *et al.* Mapping the genetic architecture of human traits to cell types in the kidney identifies mechanisms of disease and potential treatments. *Nat Genet* **53**, 1322-1333, doi:10.1038/s41588-021-00909-9 (2021).
